# Novel Molecular Targets for Hepatocellular Carcinoma

**DOI:** 10.3390/cancers14010140

**Published:** 2021-12-28

**Authors:** Beatrice Cavalluzzo, Angela Mauriello, Concetta Ragone, Carmen Manolio, Maria Lina Tornesello, Franco M. Buonaguro, Siri Amanda Tvingsholm, Sine Reker Hadrup, Maria Tagliamonte, Luigi Buonaguro

**Affiliations:** 1 Innovative Immunological Models Unit, Istituto Nazionale Tumori-IRCCS-“Fond G. Pascale”, 80131 Naples, Italy; beatrice.cavalluzzo@istitutotumori.na.it (B.C.); a.mauriello@istitutotumori.na.it (A.M.); concetta.ragone@istitutotumori.na.it (C.R.); carmen.manolio@istitutotumori.na.it (C.M.); m.tagliamonte@istitutotumori.na.it (M.T.); 2 Molecular Biology and Viral Oncogenesis Unit, Istituto Nazionale Tumori-IRCCS-“Fond G. Pascale”, 80131 Naples, Italy; m.tornesello@istitutotumori.na.it (M.L.T.); f.buonaguro@istitutotumori.na.it (F.M.B.); 3 T-Cells and Cancer, Experimental & Translational Immunology (XTI), Health Technology, Technical University of Denmark, 2800 Kongens Lyngby, Denmark; satv@dtu.dk (S.A.T.); sirha@dtu.dk (S.R.H.)

**Keywords:** hepatocellular carcinoma, tumor-associated antigens, cancer immunotherapy

## Abstract

**Simple Summary:**

HCC is a disease with highly unmet medical needs. Specific target antigens for the development of active (vaccine) and/or passive (adoptive T-cell therapy) cancer immunotherapy strategies are needed. The aim of our study was to exploit the high number of data derived from a public dataset to identify HCC-specific overexpressed proteins, leading to potential epitopes recognized by CD8^+^ cytotoxic T cells, which may share homology to viral epitopes. Circulating CD8^+^ T cells were revealed to be targeting both HCC and viral-related epitopes, suggesting the possible use in HCC-specific immunotherapies.

**Abstract:**

Hepatocellular carcinoma (HCC) is the third leading cause of death from cancer globally. Indeed, only a few treatments are available, most of which are effective only for the early stages of the disease. Therefore, there is an urgent needing for potential markers for a specifically targeted therapy. Candidate proteins were selected from datasets of The Human Protein Atlas, in order to identify specific tumor-associated proteins overexpressed in HCC samples associated with poor prognosis. Potential epitopes were predicted from such proteins, and homology with peptides derived from viral proteins was assessed. A multiparametric validation was performed, including recognition by PBMCs from HCC-patients and healthy donors, showing a T-cell cross-reactivity with paired epitopes. These results provide novel HCC-specific tumor-associated antigens (TAAs) for immunotherapeutic anti-HCC strategies potentially able to expand pre-existing virus-specific CD8^+^ T cells with superior anticancer efficacy.

## 1. Introduction

Hepatocellular carcinoma (HCC) is the fifth most common cancer and accounts for 8.2% of all cancer-related deaths globally, second only to lung cancer, with more than 800,000 new cases and deaths per year [1].

Surgery (e.g., resection and transplantation) is the most effective treatment for earlystage HCC patients from an intention-to-treat perspective, leading to a 60–80% five-year survival. In more advanced disease stages, loco-regional therapies are recommended, with dramatically lower and highly variable survival rates [2]. For many years, the tyrosine–kinase inhibitor sorafenib has been the only approved first-line systemic therapy for advanced unresectable HCC. Only recently has the inhibitor of vascular endothelial growth factor receptors 1–3, lenvatinib, also been approved. Few other drugs have been approved as second-line treatments, but overall, a very limited survival benefit is achieved unless patients are stratified according to gene expression signature [3,4,5]. Very recently, the IMBRAVE150 trial based on a combination of atezolizumab plus bevacizumab was shown to extend median progression-free survival (PFS) and overall survival at 12 months (OS), compared with sorafenib [6].

Concerning immunotherapy strategies—namely, adoptive T-cell therapies (ACTs) and therapeutic cancer vaccines, to date, very few trials have been conducted, targeting a limited number of overexpressed target cellular proteins with unsatisfactory results [7]. Among these, only the glypican-3 (GPC-3) appears to be highly specific for HCC, with high expression in tumor cells and low or no expression in normal cells [8]. Novel-shared, HCC-associated antigens have been recently identified within the HEPAVAC project and a multi-epitope, multi-HLA peptide vaccine has been evaluated in a phase I/II clinical trial in an adjuvant setting enrolling early intermediate stage HCC patients (HepaVac-101—NCT03203005) [9,10,11]. HCC-specific mutated neoantigens have been searched for personalized anticancer immunotherapy, but the number of peptides identified with high-throughput screening procedures is extremely limited, probably due to the low tumor mutational burden (TMB) characterizing liver cancer [12,13,14].

Therefore, the identification of additional proteins highly expressed in HCC with low or no expression in normal liver cells is greatly needed as diagnostic markers. Moreover, if such proteins show low or no expression in the vast majority of normal cells, they can be ideal targets for immunotherapy strategies.

In the past, global gene expression and bioinformatics analyses have been adopted by several groups to identify differentially expressed genes (DEGs) and functional pathways involved in the progression of HCC [14,15,16,17,18]. However, although a large number of genes differentially expressed in cancer tissue, compared with normal tissue, have been described, a consensus signature has not been identified yet. The only diagnostic and prognostic biomarker validated so far is alpha-fetoprotein (AFP), while osteopontin and glypican-3 are currently under investigation for the early diagnosis of HCC [19]. Such a lack of validated HCC-specific biomarkers also represents a significant limitation in the selection of potential druggable and immune targets. Thus, novel computational approaches should be developed by merging multiple datasets from different platforms to identify and validate biomarkers with significant precision and reproducibility.

To this aim, we aimed to apply an algorithm different from the ones described so far. Indeed, we started by analyzing the HCC-specific protein expression levels by interrogating the Human Protein Atlas, which contains protein and mRNA expression data from 17 different forms of human cancer (https://www.proteinatlas.org/ (accessed on 15 February 2021) [20]. Selected proteins were fully evaluated by several means, including correlation with patients’ survival, protein network analyses, as well as prediction of target epitopes for immunotherapy. Homology of the latter with virus-derived epitopes was assessed by Blast search, and an MHC class l-tetramer staining was performed to assess the frequency of CD8^+^ T-cell clones specific for the paired peptides.

## 2. Materials and Methods

### 2.1. Protein Expression Analysis

Starting from the downloadable data section of the Human Protein Atlas database (https://www.proteinatlas.org/about/download (accessed on 15 February 2021)), the Normal Tissue and Pathology datasets were downloaded. They contain expression profiles evaluated by immunohistochemistry on tissue microarrays of proteins of different human tissues samples, nontumoral and tumoral, respectively.

The normal tissue dataset is an extremely wide list, containing information about the expression of several proteins in different cell types of a specific human tissue. To identify tumor-specific proteins, only those proteins that were defined as “not detected” in hepatocytes and bile duct cells were selected. Subsequently, these proteins were searched in the Pathology dataset to select only those detected in “liver cancer” tissue samples at “high”, “medium”, or “low” expression level.

### 2.2. Survival Analysis

Survival analysis was performed taking advantage of data at the pathology section of The Human Protein Atlas (https://www.proteinatlas.org/humanproteome/pathology (accessed on 15 February 2021)). Only proteins whose high expression was correlated with poor survival rate (*p* < 0.05) were selected for subsequent analysis.

### 2.3. HCC Samples Collection and Transcriptomic Analysis

Tumor and paired nontumor liver tissues were collected from 24 HCC patients at the National Cancer Institute (INT) of Naples “G. Pascale”. Samples were stored in 0.5 mL of RNAlater stabilizing solution (Ambion, Austin, TX, USA) for 16 h at 4 °C and then transferred and preserved at −80 °C. Stored samples were used to perform an RNA-seq analysis.

The transcript levels of overexpressed proteins were also evaluated on the Gene Expression database of Normal and Tumor Tissues 2 (GENT2) database (http://gent2.appex.kr (accessed on 18 March 2021)), a search platform for gene expression patterns across different normal and tumor tissues compiled from public gene expression datasets.

### 2.4. Biological Networks and Interaction Analysis

To evaluate biological function and interactions of selected proteins, a network interaction analysis was performed on STRING (https://string-db.org/ (accessed on 19 March 2021)) [21,22]. This is a database of known and predicted protein–protein interactions, including physical and functional associations that stem from computational prediction, from knowledge transfer between organisms, and from interactions aggregated from other (primary) databases. First, was performed a “multiple proteins” analysis, including all selected proteins; setting parameters included “evidence” for the meaning of network edges, all the active interaction sources, a minimum interaction score of medium confidence (0.400), and a max “number of interactors to show” of no more than 10 for both 1st and 2nd shell. After obtaining a protein–protein interaction network, biological processes involving those proteins were evaluated (Σ Analysis). Using the same (previously described) settings parameters, except for the number of interactions (10 interactions selected only for 1st shell, none for 2nd shell), a single protein analysis was performed, and different pathways were evaluated.

### 2.5. HLA Class I Epitope Prediction

In order to predict MHC-class I epitopes, protein sequences were downloaded from UniProt database (https://www.uniprot.org/ (accessed on 23 March 2021)), and the entire sequence was analyzed with NetMHCpan4.1 tool (https://services.healthtech.dtu.dk/service.php?NetMHCpan-4.1 (accessed on 23 March 2021)), to predict the binding affinity [23] and with NetMHCstabpan1.0 tool (https://services.healthtech.dtu.dk/service.php?NetMHCstabpan-1.0 (accessed on 25 March 2021)), to predict the stability of 9-mer peptides [24]. Prediction analyses were performed for the HLA-A*02:01 and HLA-A*24:02 alleles, selecting only peptides considered weak and strong binders, according to default parameters (WB; SB).

### 2.6. BLAST Homology Search

Peptides selected as SB according to NetMHCpan 4.1 prediction tool were submitted to BLAST for a homology search against viruses (tax id: 10239) within the nonredundant protein sequences database (https://blast.ncbi.nlm.nih.gov/Blast.cgi (accessed on 26 March 2021)). Virus-derived homologous sequences were analyzed to predict their binding affinity and stability to HLA-A*02:01 and HLA-A*24:02 alleles, as described above.

### 2.7. Epitope Modeling and Molecular Docking

The 3D structure of interaction between peptides and HLA-A*02:01 was generated using Pymol software (PyMol Molecular graphics system, version 1.8.6.2) and Molsoft ICM (http://www.molsoft.com/, version 3.8-7d (accessed on 14 April 2021)) software. The PDB format of the complex between HLA-A*02:01 (1AO7), a viral peptide (TAX), and human T-cell receptor was downloaded from RCS Protein Data Bank (PDB) website (https://www.rcsb.org/structure/1AO7 (accessed on 14 April 2021)) [25]. The original viral peptide sequence was replaced with individual predicted epitopes using the Pymol visualization system, and Molsoft ICM software was used to visualize the molecular docking of HLA-A*02:01 with selected 9-mers.

### 2.8. Peptide Synthesis and Solubilization

All peptides were synthesized at a purity of ≥90% (GenScript, Piscataway, NJ, USA). Lyophilized powders were reconstituted in DMSO Solution (CARLO ERBA Reagents S.r.l., Cornaredo, Italy) and diluted in 90% of 1X PBS (HyClone, Thermo Fisher Scientific Inc., Waltham, MA, USA).

### 2.9. Peptide Binding Affinity and BFA Decay Assays

Peptide binding affinity to HLA-A*02:01 molecule and BFA decay assays were performed for each candidate peptide. Human TAP-deficient T2 cell line (174xCEM.T2; ATCC CRL 1992™) was purchased from American Type Culture Collection (ATCC; https://www.atcc.org/ (accessed on 3 March 2021)) and cultured in Iscove’s modified Dulbecco’s medium (IMDM; Gibco Life Technologies, Thermo Fisher Scientific Inc., Waltham, MA, USA) containing 25 mM HEPES and 2 mM L-glut, supplemented with 20% fetal bovine serum (FBS; Capricorn Scientific GmbH, Ebsdorfergrund, Germany), 100 IU/mL penicillin and 100 μg/mL streptomycin (Gibco Life Technologies). Cells were maintained at 37 °C in a humidified incubator with 5% CO_2_. Briefly, T2 cell lines were seeded at 3.5 × 10^5^ cells per well in 24-well plates and incubated for 16 h at 27 °C with peptides (final concentrations: 5, 10, 20, 50, and 100 μM) in IMDM serum-free medium. The next day, cells were incubated for an additional 2 h at 37 °C. Following incubation, cells were harvested and centrifuged at 200× *g* for 5 min. Subsequently, cells were washed twice with phosphate-buffered saline (1X PBS; Gibco Life Technologies) and stained with R-PE conjugated anti-human HLA-A2 monoclonal antibody (cat. 343306; BioLegend, San Diego, CA, USA), for 30 min at 4 °C, and analyzed with the Attune™ NxT flow cytometer (Thermo Fisher Scientific). Mouse H-2Kb-specific OVA SIINFEKL peptide was used as a negative control, and T2 cells without any added peptide were used as a background control. HLA-A*02:01 MUC1 LLLLTVLTV peptide was used as a positive control. A fluorescence index (FI) was calculated using the following formula: FI = mean fluorescence intensity (MFI) sample—MFI background]/MFI background, where MFI background represents the value without peptide. A value of FI > 0.5 was set as a threshold to indicate peptides with affinity for the HLA-A*02:01 molecule. For the brefeldin A decay assay, T2 cells were incubated with peptides (50 μM) as described above, washed, and treated with 1X BFA (brefeldin A solution; cat. 420601; BioLegend) in IMDM serum-free medium, for 1 h at 37 °C. Cells were harvested every two hours (T0, T2, T4, T6, T8), washed with phosphate-buffered saline (1X PBS; Gibco Life Technologies), stained with anti-HLA-A*0201 fluorescent monoclonal antibody (cat. 343306; BioLegend), and analyzed by flow cytometry. The stability of each peptide bound to HLA-A2 was measured as the DC_50_ value, which was defined as an estimate of the time required for a 50% reduction in the MFI value at time 0. The DC_50_ value was calculated according to the following formula: MFI at indicated time points/MFI at time 0 × 100. All the experiments were performed in triplicate.

### 2.10. pMHC Multimer Preparation and T-Cell Staining

pMHC complexes were generated by combining purified disulfide-stabilized HLA A*0201 monomer (100 μg/mL) with 100 μM peptide for 30 min in PBS at room temperature [26]. Then, pMHC complexes were centrifuged for 5 min at 3300× *g* to sediment any aggregated MHC molecules. For each 100 μL pMHC, 9.02 μL (0.2 mg/mL stock, SA-PE (BioLegend, 405204), SA-APC (BioLegend, 405207), SA-PE/Cy7 (BioLegend, 405206), SA-PE-CF594 (BD, 562284)), or 18.04 μL (0.1 mg/mL stock, SA-BV421 (BioLegend, 405226), SA-BV650 (BioLegend, 405231) streptavidin conjugate was added and incubated for 30 min on ice. D-biotin (Sigma-Aldrich, St.Louis, MO, USA) was added at a final concentration of 25 μM to block any free binding sites and multimers were stored at −20 °C with 5% glycerol and 0.5% BSA. To stain for T-cell reactivity, PBMCs from 4 HCC patients and 3 healthy donors (2–5 × 10^6^) were incubated with a pool of pMHC multimers (3 μL/multimer) and dasatinib (50 nM final, LC laboratories, D-3307) for 15 min at 37 °C. Cells were then stained with antibodies CD3-FITC (1:40, BD, 345763) and CD8-BV480 (1:100, BD, 566121) and LIVE/DEAD Fixable Near-IR (1:1000, Invitrogen, Waltham, MA, USA L10119) for 30 min on ice and washed twice in FACS buffer (PBS + 2% FCS). Gating for CD3^+^/CD8^+^ T cells was performed on live cells, and binding to pMHCs was assessed by measuring specific fluorescence associated with each individual pMHC. Samples were acquired on a flow cytometer (LSRFortessa, BD, Franklin Lakes, NJ, USA).

### 2.11. Statistical Analysis

Comparisons between individual data points were performed with the two-sided Student’s *t*-test and ANOVA, as appropriate. The comparisons were paired for the 24 matched samples collected at the INT and unpaired for the samples extracted by the publicly available data (http://gent2.appex.kr/gent2/ (accessed on 18 March 2021)). Normally distributed data were represented as mean ± S.E.M. Two-way ANOVA and Bonferroni post hoc analysis were used to examine the significance of differences among groups. All *p* values were two tailed and considered significant if less than 0.05.

## 3. Results

### 3.1. Identification of HCC-Related Proteins

**Figure 1:**
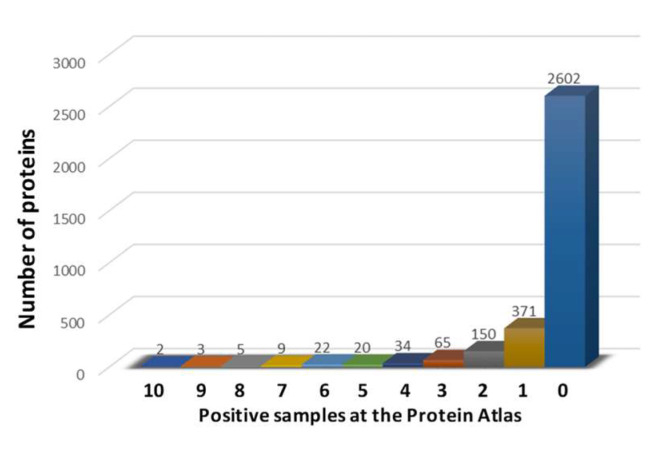
Number of proteins expressed in liver tumor samples, divided by the number of positive samples with high level of expression.

In order to identify proteins specifically overexpressed in HCC and not detected in normal liver cells, the Normal Tissue dataset, available at the Human Protein Atlas (https://www.proteinatlas.org/about/download (accessed on 15 February 2021)), was searched. More than 15,000 proteins were screened, and 5446 of them were found not detected in hepatocytes and bile duct cells of normal liver. In order to evaluate if and which of those proteins were expressed in liver tumors, the Pathology dataset was analyzed, and a total of 3283 proteins were found to be expressed in the liver tumor samples, at different levels. In particular, only a discrete number of such proteins showed high expression in more than one sample of the Protein Atlas (310, 9.45%), while the vast majority of them showed high expression in one or no sample at all (2973, 90.55%) (Figure 1). To verify the tumor-specificity of such proteins, the Normal Tissue dataset was reexamined to exclude the expression of these 310 proteins in all other normal cells. The analysis identified 40 proteins with different expression levels in HCC that were absent in all normal tissues (HCC-specific proteins = HSP). Of these, 16 proteins were found to be expressed by immunohistochemistry (IHC) in all HCC samples at the Human Protein Atlas database, with a medium/high expression in 87.17% of samples on average (min 50%–max 100%). The remaining 24 proteins were not detected by IHC in a percentage of HCC samples, ranging from 8.33% (1/12) to 50% (6/12) (Table S1).

### 3.2. Survival Analysis

**Figure 2:**
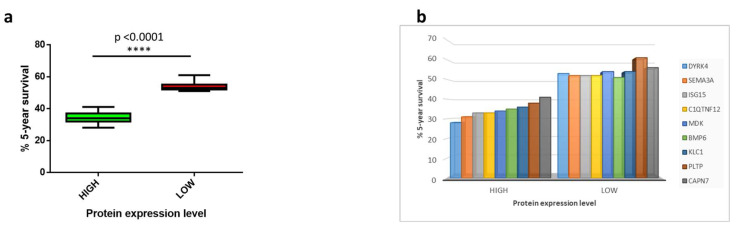

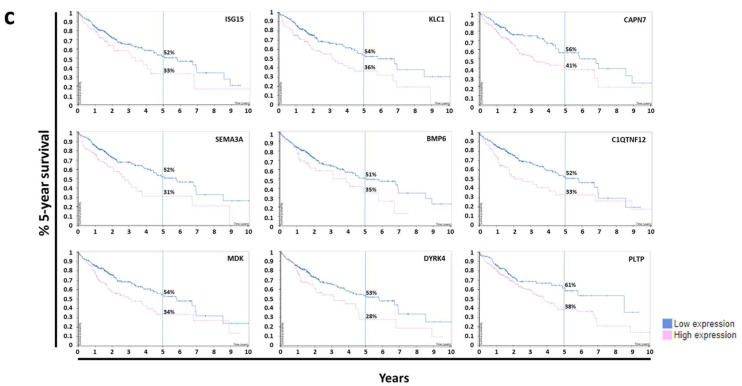
Survival analysis: (**a**) the % 5-year survival average in HCC patients with high or low expression level of all 9 HSP-pp; (**b**) chart showing % 5-year survival for each of the selected proteins related to their level of expression; (**c**) Kaplan–Meier curves for the 9 HSP-pp.

Survival analysis was performed for each of the selected 40 HSPs in order to identify those which are associated with an unfavorable prognosis in HCC patients (*p* < 0.05). A total of nine proteins were identified with such a characteristic (HSP associated with poor prognosis = HSP-pp) (Table 1). The five-year survival in HCC patients with high expression of each of the nine HSP-pp ranged from 28% to 41%. Considering the high expression of all nine HSP-pp, the average five-year survival in HCC patients was 34.33%, compared with 53.88% in HCC patients with low expression (Figure 2a). In particular, the worst prognosis was associated with the high expression of DYRK4 protein, with a delta of 25 percentage points (28% vs. 53% at low expression). On the other extreme, the best prognosis was associated with the high expression of CAPN7 protein, with a delta of 15 percentage points (41% vs. 56% at low expression) (Figure 2b,c).

### 3.3. Gene Expression Analysis

**Figure 3:**
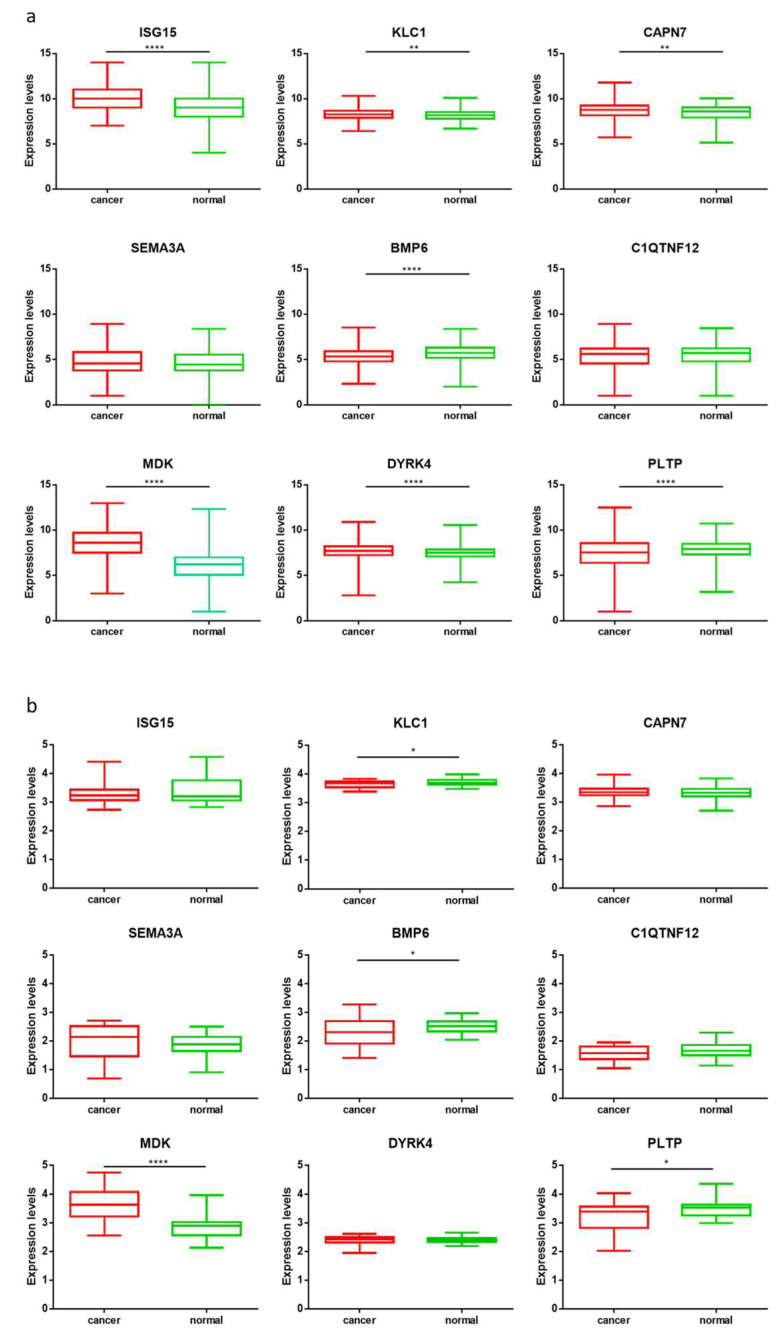
Gene expression levels in tumoral and nontumoral liver tissue: (**a**) transcript levels of 9 HSP-pp from 24 HCC patients; (**b**) transcript levels of 9 HSP-pp from GENT2 database. *p* < 0.0001 (****); *p* < 0.05 (**); *p* < 0.01 (*).

The gene expression of the selected nine HSP-pp was assessed by RNASeq analysis performed on paired HCC samples and matched nontumoral liver tissues from 24 HCC patients (Table S2). The results showed a trend of a higher expression level in cancer tissues for ISG15, CAPN7, SEMA3A, and DYRK4 genes. The MDK gene showed the most relevant increased expression in cancer tissues with a *p* value < 0.0001. On the contrary, KLC1, BMP6, C1QTNF12, and PLTP genes showed higher expression levels in the normal tissues. In order to confirm such results on a much larger dataset, we took advantage of publicly available data (http://gent2.appex.kr/gent2/ (accessed on 18 March 2021)). Gene expression levels from 691 liver cancer tissues were compared with 297 normal tissues. The analysis confirmed the data generated on the 24 HCC samples of our cohort and was supported by a high statistical *p* value, except for SEMA3A and C1QTNF12 genes (Figure 3a,b). The gene expression levels showed a significant correlation with the protein expression reported at the Protein Atlas for most of the selected HSP-pp (data not shown).

### 3.4. Analysis of Pathways

The pathway analysis showed that for some of the selected HSP-pp, the involvement in biological processes was directly associated with tumor evolution. In particular, SEMA3A was involved in cell migration, BMP6 in transcription and cell differentiation, and MDK in cell migration, and apoptosis processes. Others were involved in processes related to either the hepatitis viral infection, such as the type I interferon signaling (ISG15) pathway, or the physiological liver functions, such as plasma lipoprotein particle (PLTP) organization (Table S3; Figures S1–S9).

The analysis of protein–protein interactions showed a lack of direct interaction between the nine proteins, and only ISG15, SEMA3A, and MDK had a common interactor in STAT1 (Figure S10). However, few biological processes were identified including more than one of the nine proteins, in particular processes related to response to external stimuli, receptor signaling pathway, cell motility and migration, and apoptosis. All of them were supported by extremely low false-discovery rate (FDR), ranging from 2.04 × 10^−10^ (defense response, GO:0006952) to 1.91 × 10^−2^ (regulation of apoptosis process, GO:0042981). ISG15, SEMA3A, BMP6, and MDK were the proteins most frequently involved together in such biological processes (Figure S11).

### 3.5. Peptide Prediction

Anticancer CD8^+^ T cells are elicited by nine aa-long peptides presented by the major histocompatibility complex (MHC) class-I. Therefore, in order to screen potential MHC class I-associated tumor-associated antigens (TAAs) from the HSP-pp, the NetMHCpan software was used to predict nine aa-long epitopes (nonamers) associated with the HLA-A*02:01 and 24:02 alleles. The analysis was performed considering all the overlapping nonamers with a one-amino-acid lateral shift covering the entire protein sequence. Only a discrete number of strong (SB) and weak (WB) binders to the two alleles were predicted for each protein. Overall, considering both alleles, 99 SBs and 221 WBs were predicted. In particular, the MDK protein showed the lowest (3 SB and 2 WB), and the CAPN7 protein showed the highest (25 SB and 47 WB) cumulative number of binders (Table S4).

**Figure 4:**
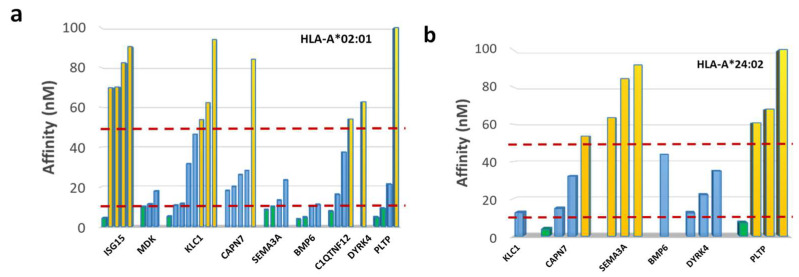
Number of epitopes with affinity > 50 nM, 10 nM < affinity < 50 nM and affinity < 10 nM for (**a**) HLA-A*02:01 and (**b**) HLA-A*24:02.

Among the 99 SBs, only those with a predicted affinity lower than 100 nM were selected and further subdivided into three groups, characterized by affinity < 10 nM, 10 < affinity < 50 nM, affinity > 50 nM. Indeed, only those with a predicted affinity <100 nM have been previously shown to have a 100% concordance with ex vivo binding assay [27]. According to such a classification, 36 SBs were selected in both HLA-A alleles, of which 12 peptides with affinity < 10 nM and 24 peptides with 10 < affinity < 50 nM (Figure 4a,b).

Subsequently, the NetMHCstabpan software was used to implement the prediction analysis with information on the binding stability of the peptides to the HLA molecule, expressed as half-life time in hours (Thalf). According to this combined analysis, only 28 predicted epitopes were classified as SB, with a Thalf ranging from 1.96 h (the HLA-A*24:02 linked peptide VHMKDFFYF from the DYRK4 protein) to 61.9 h (the HLA-A*24:02 linked peptide VYSACSFTF from the CAPN7 protein). Therefore, the number of SBs per protein was, on average, 3.11, ranging from 1 (ISG15, MDK, and C1QTNF12) to 6 (CAPN7 and PLTP) (Table S5). In order to verify whether the predicted peptides have been already identified in normal tissues, the HLA Ligand Atlas (https://hla-ligand-atlas.org/welcome (accessed on 13 April 2021)) was searched. Among the 28 SB predicted by our analysis in the 9 HCC proteins, only 4 have been already identified by mass spectrometry as HLA allele-specific HLA ligands in normal tissues (Table S6).

### 3.6. Identification of Homologous Viral Epitopes

We have recently shown that TAAs may share high homology to viral antigens, suggesting an established antiviral memory T-cell immunity able to cross-react with tumor antigens [28]. Therefore, the predicted 28 SBs were further screened for homology to sequences derived from human viruses in BLAST, and several viral peptides were found. However, only four of them were predicted to be strong binders to HLA-A*02:01 with a Thalf > 3 h (Table S6). Of these, the predicted MLAGNAFTA epitope derived from the human calicivirus showed 7/9 identical residues, with the MLAGNEFQV epitope from the ISG15 protein. The predicted ALMAFTSAV epitope derived from the HCV and the LLLTLLLLL epitope derived from the human adenovirus 4 showed 7/9 and 8/9 identical residues, with the ALLALTSAV and LLLTLLALL epitopes from the MDK protein, respectively. Finally, the predicted LLGPLLVLL epitope derived from the HBV showed 8/9 identical residues, with the LLGPQLVLL epitope from the C1QTNF12 protein. Strikingly, the epitope derived from the HCV showed affinity and stability to the HLA-A*02:01 significantly higher than the corresponding TAA (3.11 vs. 10.06 nM; 45.58 vs. 9.85 h) (Table 2).

Likewise, only three viral peptides sharing high homology with those derived from the HCC proteins were predicted to bind the HLA-A*24:02 molecule. However, the affinity was much lower (>70 nM) and the stability was <1.5 h (Table 2).

### 3.7. Peptide Modeling and Molecular Docking

In order to verify that predicted paired HCC-TAA and viral epitopes share similar contact residues with both the HLA molecule and the TCR, epitope modeling and molecular docking were performed.

Epitopes crystallized with the HLA-A*02:01 and the TCR showing sequence homology with TAA peptides were not found. Therefore, the 1AO7 crystallized complex including the HTLV-I TAX epitope was used as a general template to conduct the analyses. All four paired TAA and viral epitopes shared identical contact patterns with the HLA molecule and extremely similar contact patterns with the β chain of the TCR. On the contrary, the contact patterns with the α chain of the TCR showed narrow differences in the paired epitopes, which were most obvious in the paired MDK (LLLTLLALL)–human adenovirus (LLLTLLLLL) peptides. Indeed, the substitution of Ala with a Leu in p7 induced a significant modification in the peptide portion interacting with the α chain of the TCR due to the lateral chain of the Leu amino acid (Figure S12).

**Figure 5:**
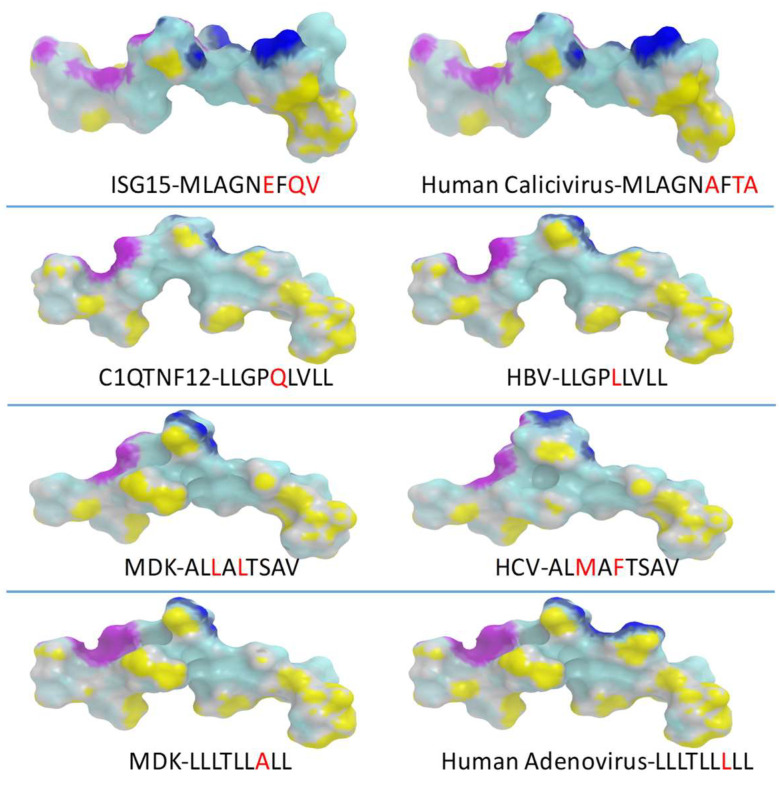
Predicted 3D conformation of paired peptides. The conformation of the TAA peptides and paired viral antigens bound to the HLA-A*02:01 molecule is shown. The prediction was performed using as template structure the HTLV-I LLFGYPVYV peptide crystallized with the HLA-A*0201 molecule, the β2 microglobulin, the α and β chains of the T-cell receptor (TCR) (PDB https://www.rcsb.org/structure/1AO7 (accessed on 14 April 2021)). Yellow areas = contact points with HLA molecule; blue areas = contact points with the TCR α chain; violet areas = contact points with the TCR β chain.

This would suggest that the β chains of the TCR clones induced by the viral epitopes would perfectly interact with the corresponding portion of the paired TAA peptides, while the binding affinity of the coupled α chains would change according to the more or less pronounced differences observed in the contact patterns (Figure 5).

On the contrary, due to the lack of crystallized structures deposited in the PDB of nonamers including both HLA and TCR for HLA-A*24:02 allele, the paired HLA-A*24:02 restricted TAA and viral epitopes could be compared only for their interaction with the HLA molecule, showing extremely similar contact patterns (Figure S13). Nevertheless, the conformation of the bound structures was highly suggestive of a similar contact pattern with both the α and β chains of the TCR. The exception was found to be the DYRK4 (FYFRNHFCI)–influenza virus (DYFRNQFKI) paired peptides, which showed a dramatic difference in the portion in contact with the α chain. Indeed, the substitution of the His with a Gln in p6 and the Cys with a Lys in p8 could likely induce a significant modification in the peptide portion interacting with the α chain of the TCR due to the lateral chains of the changed amino acids (Figure S14).

### 3.8. In Vitro Analysis of Binding Affinity and Stability to HLA-A*02:01 Molecule

**Figure 6:**
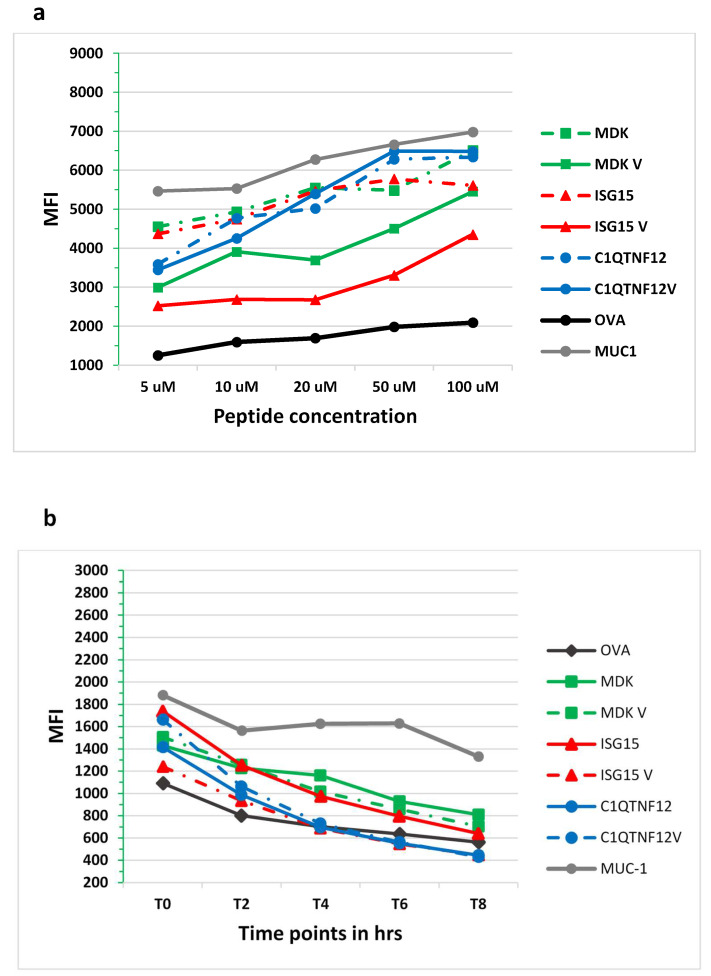
Experimental binding of TAAs and viral paired peptides to HLA-A*0201. Binding to HLA-A*0201 molecule and relative stability was assessed in TAP-deficient T2 cells loaded with the indicated peptides: (**a**) mean fluorescence intensity at flow cytometer indicates binding levels of peptides to HLA molecules; (**b**) decay of mean fluorescence intensity over time indicates stability of the peptide binding to the HLA molecule.

In order to experimentally confirm the binding and stability of peptides to the HLA-A*02:01 molecule, the TAP-deficient HLA-A*02:01-positive T2 cell line was loaded with the individual peptides from each pair. The analysis confirmed that each peptide bound the HLA-A*02:01 molecule induced a dose-dependent increase in the HLA surface expression on T2 cells over the background, as well as the OVA negative control level. The highest level of binding was observed for the paired C1QTNF12 and HBV peptides, characterized by the same MFI. For the other two pairs, the binding of the TAA was clearly stronger than the paired viral epitope. Overall, the binding assay confirmed the high affinity of all peptides to the HLA-A*02:01 molecule as suggested by the prediction analysis (Figure 6a). The peptide–MHC dissociation kinetics showed that the 50% dissociation value (Thalf) was reached 4 h after the peptide loading (T4), for the ISG15 and C1QTNF12 pairs, while it was never reached up to 8 h after the peptide loading (T8) for the MDK pair (Figure 6b). Strikingly, the observed Thalf for each peptide was in agreement with the predicted values.

### 3.9. Detection of Epitope-Specific CD8^+^ T-Cell Clones

**Figure 7:**
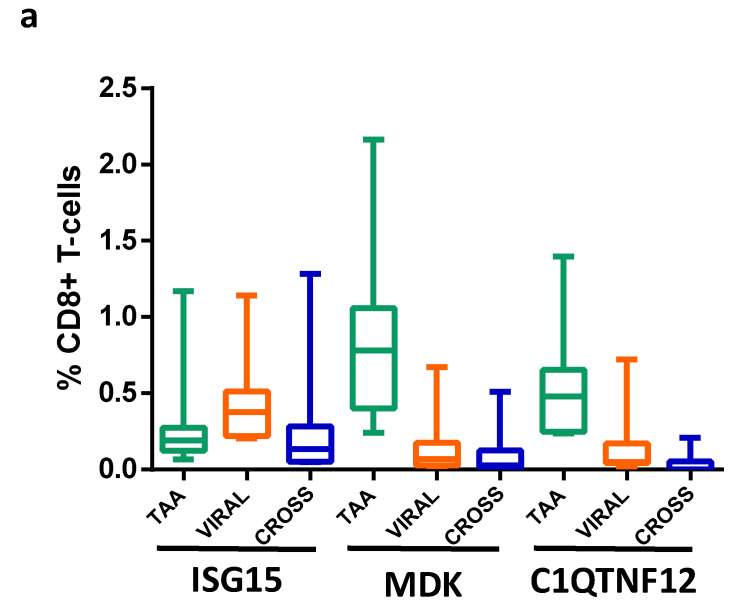

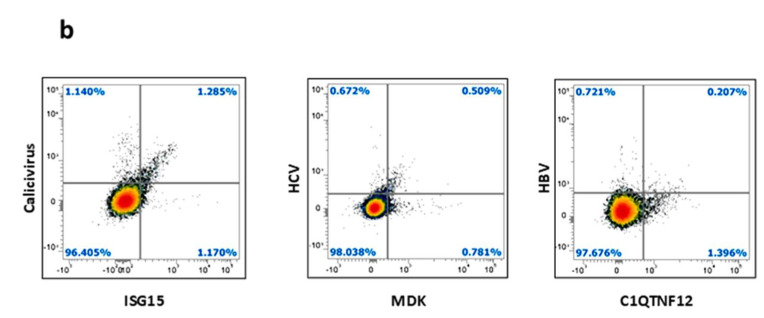
Evaluation of epitope-specific CD8^+^ T-cell clones recognizing TAA epitope, VIRAL paired epitope, and both peptides (CROSS): (**a**) cumulative data from HCC and healthy donors are shown. (**b**) pMHC-directed expansion of T cells validated the presence of T cells specific for each paired TAA and viral epitopes in a single subject. Frequencies of pMHC multimer-specific T cells out of total CD3^+^ CD8^+^ are displayed.

In order to confirm the antigenicity of the predicted peptides, we performed an analysis to detect CD8^+^ specific T-cell clones in PBMCs from HCC patients and healthy subjects. MHC-class I tetramers for HLA-A*02:01 were loaded with individual peptides from ISG15 (*MLAGNEFQV*), MDK (*ALLALTSAV*), and C1QTNF12 (*LLGPQLVLL*). The results showed the presence of circulating CD8^+^ T cells specific for all three peptides in both HCC and healthy subjects, although at a significantly different level (Figure 7a; Supplementary Figure S15). In particular, the percentage of CD8^+^ T cells reacting with the MDK peptide was the highest (average 0.88%), followed by C1QTNF12 (0.55%) and ISG15 (0.32%). In parallel, the T-cell reactivity against the paired viral-derived epitopes showed a different pattern. Indeed, the highest percentage of reactivity was observed for the human calicivirus *MLAGNAFTA* peptide homologous to ISG15 (average 0.46%), followed by the hepatitis C virus *ALMAFTSAV* peptide homologous to MDK and the hepatitis B virus *LLGPLLVLL* peptide homologous to C1QTNF12 (average 0.16%). The T-cell cross-reactivity against the paired epitopes directly reflected the reactivity against the viral-derived epitopes, with the highest percentage of cross-reactive cells against the ISG15 pair (average 0.298%), followed by the MDK pair (average 0.11%) and the C1QTNF12 pair (average 0.04%) (Figure 7a,b).

## 4. Discussion

In the present study, we aimed at identifying novel molecular targets for hepatocellular carcinoma (HCC), a disease with highly unmet medical needs. The overall strategy pursued in the study is shown in Supplementary Figure S16.

Protein identification. The Human Protein Atlas (https://www.proteinatlas.org/ (accessed on 15 February 2021)) was interrogated and 3283 proteins were found to be expressed at different levels in the liver tumor samples. Of these proteins, only 310 showed high expression in more than one sample, and only 40 proteins were not expressed in any normal tissues. Furthermore, survival analyses showed a statistically significant association between overexpression in HCC patients and poor prognosis for only nine of these proteins. Consequently, the latter were selected as HCC-specific targets for subsequent analyses given that they are overexpressed only in multiple HCC samples, not expressed in normal tissues and associated with poor prognosis. RNASeq analysis generated on HCC samples experimentally collected at our Institute and validated by data at publicly available databases confirmed that all the selected nine genes, except for SEMA3A and C1QTNF12, were significantly overexpressed in HCC samples, compared with normal tissues. The pathway analysis showed the involvement of the selected proteins in biological processes directly associated with tumor evolution (SEMA3A, BMP6, MDK), hepatitis viral infection (ISG15), or physiological liver functions (PLTP).

For epitope prediction, in order to identify potential novel epitopes as the target for innovative HCC-specific immunotherapy strategies, the NetMHCpan software was used to predict binders to the MHC class-I HLA-A*02:01 and 24:02 alleles, which altogether cover about 40% of the world population. In particular, about 45% of European and North American Caucasian populations, 40% of the Chinese population, and 20% of the Indian population (http://www.allelefrequencies.net (accessed on 19 March 2021)). Among the predicted strong binders (SBs), only those with a very high affinity (<100 nM) were selected for the present analysis, given that, according to our previous studies, only these are confirmed to bind the HLA-A*02:01 molecules in the TAP-deficient T2 cell line experimental method in 100% of the cases [24]. According to these parameters, 58 SBs were predicted for the two HLA-A alleles (42 HLA-A*02:01, 16 HLA-A*24:02). Of these, 12 peptides (10 HLA-A*02:01, 2 HLA-A*24:02) showed a predicted affinity <10 nM, and 24 peptides (17 HLA-A*02:01, 7 HLA-A*24:02) an affinity between 10 and 50 nM. The remaining 22 peptides showed an affinity between 50 and 100 nM (15 HLA-A*02:01, 7 HLA-A*24:02). When the stability values (expressed as Thalf) were included in the analysis, a subgroup of 28 SBs with affinity <100 nM were predicted (15 HLA-A*0201, 13 HLA-A*2402), representing the best candidate for the development of immunotherapy strategies. Indeed, only epitopes with high affinity and persisting presentation to the immune system had the highest chance to elicit the strongest anticancer T-cell immune response. In particular, CAPN7 and PLTP showed the highest number of SBs (nr. 6), while ISG15, MDK, and C1QTNF12 showed the lowest number (nr. 1). Homology analysis was performed to identify homology with viral epitopes, given that we have recently shown that such homology could represent a selective advantage in controlling tumor establishment and progression. Indeed, memory T cells induced by a previous viral infection could be promptly recalled to expand by tumor antigens, showing high homology with the viral antigens [28,29]. Out of the 28 SBs, 5 showed homology with viral epitope sequences including influenza virus, hepatitis C virus (HCV), hepatitis B virus (HBV), adenovirus, human cytomegalovirus (HCMV), and human calicivirus. Those restricted to HLA-A*02:01 showed a very high affinity (<20 nM) and stability >3 h, with a peak of 45.48 h for the HCV epitope homologous to the MDK epitope.

The epitope modeling confirmed that the paired TAA and viral epitopes also shared the same contact patterns when docked into the HLA-A*02:01 molecules. In particular, conservative changes at specific positions of the paired epitopes are always predictive of structural preservation. In most cases, the paired peptides showed similar contact patterns with the TCR α and β chains, strongly suggesting that the same CD8^+^ T-cell clone may be able to cross-react with both peptides when presented in the context of the HLA-A*02:01 molecules.

As regards epitope validation, ex vivo binding assays in TAP-deficient T2 cells confirmed the reliability of the predictive algorithms, and the immunogenicity of the selected TAA and viral-derived peptides was further confirmed by tetramer binding assay, with PBMCs from both HCC patients and healthy individuals. Moreover, cross-reactive T-cell responses against the paired peptides were observed, suggesting that specific CD8^+^ T-cell clones are able to recognize similar epitopes. This further supported the idea that viral antigens may represent a “natural” anticancer preventive immunization able to elicit antiviral memory CD8^+^ T cells promptly, expanded by cancer cells expressing a homologous TAA. This would suggest that the resulting effector CD8^+^ cytotoxic T cells could possibly target and kill the cancer cells in the earliest phases of tumor development, containing the progression to later stages. As predicted, the highest percentage of CD8^+^ T cells reactive to a viral epitope and, consequently, cross-reactive against the paired epitope was observed for the human calicivirus peptide. Indeed, viruses belonging to the Caliciviridae family are one the most frequent causes of acute gastroenteritis [30]. Consequently, such human calicivirus peptide might represent a valid target antigen for immunotherapy strategies in liver cancer.

## 5. Conclusions

In conclusion, the present study described the identification of novel shared HCC-specific target antigens for the development of active (vaccine) and/or passive (adoptive T-cell therapy) cancer immunotherapy strategies for HCC, a disease with highly unmet medical needs. These target antigens would have clinical application relevance on a broad scale because they are independent of mutations that are, almost exclusively, patient specific. Interestingly, some of them showed a high sequence and structural homology with viral sequences, providing a twofold advantage: they circumvented a possible immune tolerance against cellular-derived TAAs and elicited a swift and potent cross-reactive T-cell response deriving from the immunological memory induced by the previous encounter with viral agents.

Further studies on a larger cohort of HCC patients, with different risk factors, will provide confirmation of the described findings.

This ultimately provides a novel set of antigens for developing a therapeutic anti-HCC vaccine strategy with superior potency.

## Figures and Tables

**Table 1:** HSPs associated with poor prognosis (HSP-pp). The listed proteins from Table 1 are associated at a statistical significance with poor prognosis in HCC patients.

PROTEIN	Best Expression Cut off-p Score
ISG15	0.041
KLC1	0.0012
CAPN7	0.0022
SEMA3A	0.0029
BMP6	0.0016
C1QTNF12 (FAM132A)	0.0027
MDK	0.0057
DYRK4	0.020
PLTP	0.017

**Table 2:** List of TAA and viral-derived paired epitopes predicted as strong/weak bind level (SB/WB) by both NetMHCpan4.1 and NetMHCstabpan1.0 server.

HLA	Protein	Sequence	ViralProtein	AFF	Thalf	Binding
A*02:01	**ISG15**	MLAGNEFQV		4.35	5.22	SB
		MLAGNA FTA	capsid protein human calicivirus Seq ID: AAL18874.1	13.86	3.53	WB
					
	**MDK**	ALLALTSAV		10.06	9.85	SB
		ALMAFTSAV	polyprotein HCV seq ID: AID60264.1	3.11	45.58	SB
					
	MDK	LLLTLLALL		11.34	4.69	WB
		LLLTLLLLL	E3 14.5 kDa protein human adenovirus E4 Seq ID: AGT51280.1	17.75	4.41	WB
					
	**C1QTNF12**	LLGPQLVLL		37.45	2.61	WB
		LLGPLLVLL	S protein HBV Seq ID: AUF41974.1	20.91	3.38	WB
						
A*24:02	**CAPN7**	VYSACSFTF		3.83	61.9	SB
		GSPACTFTF	protein UL29 CMV Seq ID: AFR55693.1	658.27	0.65	WB
					
	**CAPN7**	IYTVSSFSI		14.92	5.44	SB
		SYTVSSFQV	nonstructural protein 1 influenza A virus Seq ID: QEM33605.1	352.63	1.06	WB
					
	**DYRK4**	FYFRNHFCI		12.53	22.36	SB
		DYFRNQFKI	polymerase basic protein 2 influenza A virus Seq ID: AAV33795.1	77.75	1.39	WB

## Data Availability

Data and material are available at https://doi.org/10.5281/zenodo.5801248.

## Supplementary Materials

The following supporting information can be downloaded at: https://www.mdpi.com/article/10.3390/cancers14010140/s1, Table S1: HCC-specific proteins (HSP). The listed proteins are expressed at different levels only in HCC samples and no expression is reported in any normal tissue, Table S2: Risk factors and clinical stage of HCC patients enrolled for the transcriptomics analyses, Table S3: STRING analysis for each of the 9 HSP-pp. The most relevant pathways, with a very low false discovery rate value are listed, Table S4: Number of strong (SB) and weak (WB) binders predicted for the 9 HSP-pp for HLA-A*02:01 and 24:02 alleles, Table S5: List of predicted epitopes implemented with prediction of binding stability expressed as half life time in hours (Thalf), Table S6: Outcome of interrogation of the HLA Ligand Atlas for the identification by mass spectometry of the predicted peptides in normal tissues. Figures S1–S9: STRING pathway analysis for each of the 9 selected HSP-pp. Each of the shells represents one protein, and each color indicates a specific pathway in which the same protein is involved, according to the color code expressed below. Colored lines between the proteins indicate the various types of interaction evidence, as indicated in the legend, Figure S10: Evaluation of direct interaction between the 9 HSP-pp. The STRING interaction network shows the lack of direct interaction between any of the selected proteins. The only indirect connection is found between ISG15 and MDK that interact through the STAT1 protein, Figure S11: Network analysis involving more than one of the selected HSP-pp. All the biological processes examined showed a very low false-discovery rate, Figure S12: Molecular docking of MDK-TAA and human adenovirus paired HLA-A*02:01 epitopes. Outlined areas show the single aminoacidic substitution that modifies contact patterns with the α chain of the TCR molecule, Figure S13: Predicted 3D conformation of paired peptides. The conformation of the TAA peptides and paired viral antigens bound to the HLA-A*24:02 molecule, Figure S14: Molecular docking of DYRK4-TAA and influenza virus paired HLA-A*24:02 epitopes. Outlined areas show the aminoacidic substitutions that modify the 3D structure of the peptide in the portion facing the TCR molecule. Figure S15: Evaluation of epitope-specific CD8^+^ T-cell clones recognizing TAA epitope, VIRAL paired epitope, and both peptides (CROSS). Data from HCC and healthy donors are shown, Figure S16: Pipeline applied in the study. Strategy for selection of HCC-specific proteins, epitope prediction, and validation.
