# Supplementary material for: Novel Molecular Targets for Hepatocellular Carcinoma
Source: Cancers (Basel). 2021 Dec 28;14(1):140. doi: 10.3390/cancers14010140 (PMC8750630; doi:10.3390/cancers14010140)
Supplement: Supplementary file 1 [file cancers-14-00140-s001.zip › cancers-1482530-supplementary.pdf]

Supplementary Figure S1

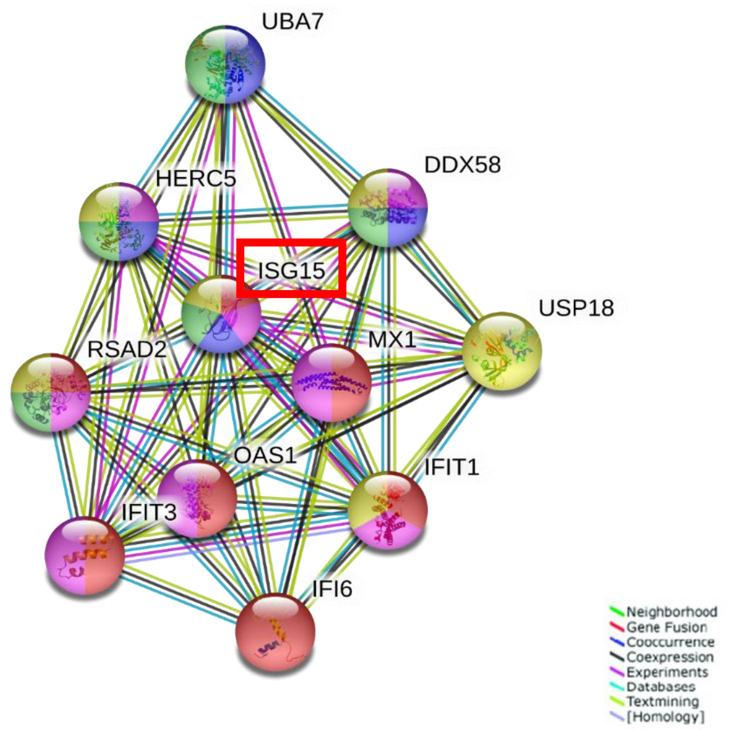

Biological Process (GO)

GO-term	description	false discovery rate	
GO:0060337	type I interferon signaling pathway	1.07e-12	
GO:0051607	defense response to virus	1.27e-12	
GO:0032480	negative regulation of type I interferon production	2.30e-07	
GO:0001817	regulation of cytokine production	0.00016	
GO:0002682	regulation of immune system process	0.00049	

Supplementary Figure S2

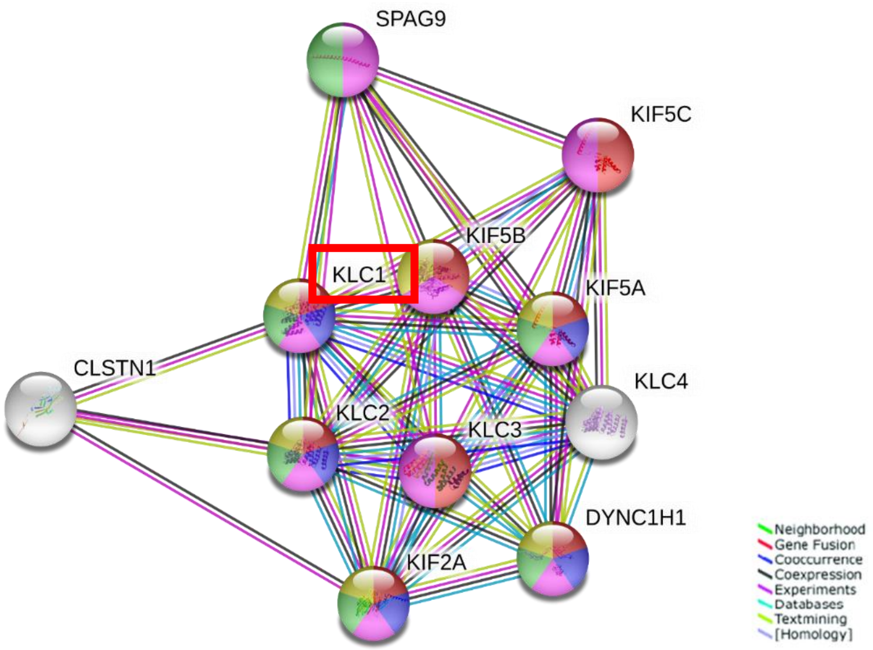

GO-term	description	false discovery rate	
GO:0007018	microtubule-based movement	1.31e-10	
GO:0019886	antigen processing and presentation of exogenous peptide antigen via MHC class II	2.30e-07	
GO:0006810	transport	0.00053	
GO:0016192	vesicle-mediated transport	0.0019	
GO:0002376	immune system process	0.0086	

Supplementary Figure S3

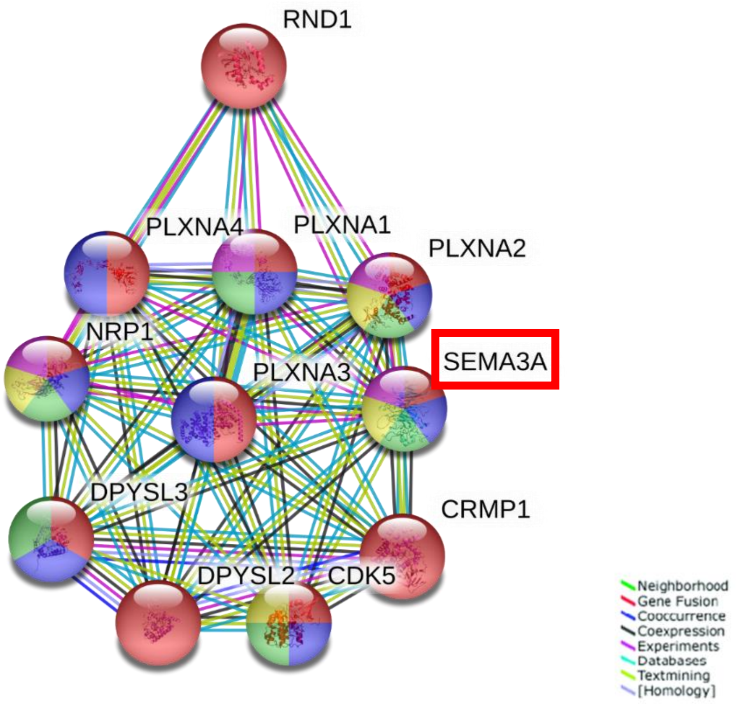

GO-term	description	false discovery rate	
GO:0048666	neuron development	4.0e-14	red
GO:0051270	regulation of cellular component movement	3.14e-08	blue
GO:0030334	regulation of cell migration	9.33°-06	green
GO:0016477	cell migration	0.0033	purple
GO:0060429	ephitelium development	0.0080	yellow

Supplementary Figure S4

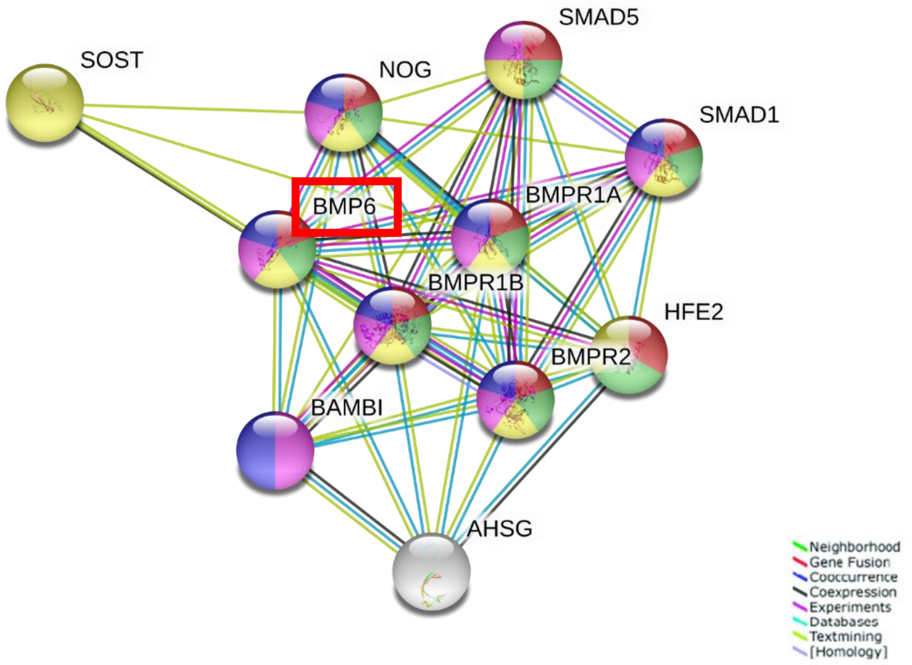

GO-term	description	false discovery rate	
GO:0030509	BMP signaling pathway	3.45e-14	
GO:0045944	positive regulation of transcription by RNA polymerase II	4.31e-07	
GO:0007166	cell surface receptor signaling pathway	2.60e-06	
GO:0045595	regulation of cell differentiation	6.97e-06	
GO:0042127	regulation of cell population proliferation	5.71e-06	

Supplementary Figure S5

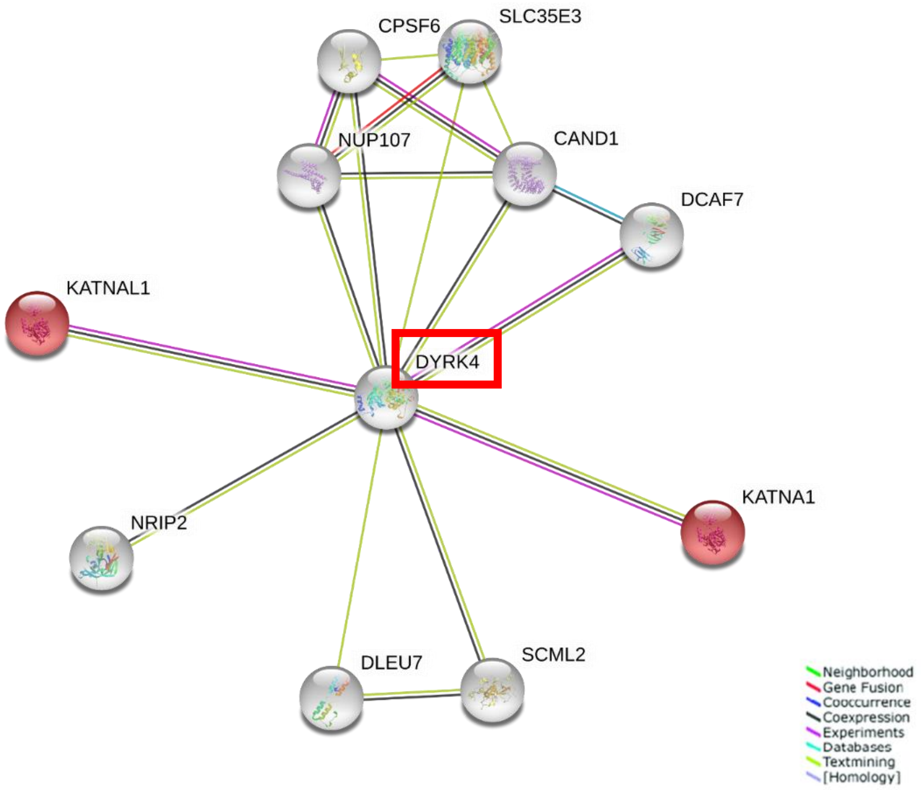

GO-term	description	false discovery rate
GO:051013	microtubule severing	0.0026

Supplementary Figure S6

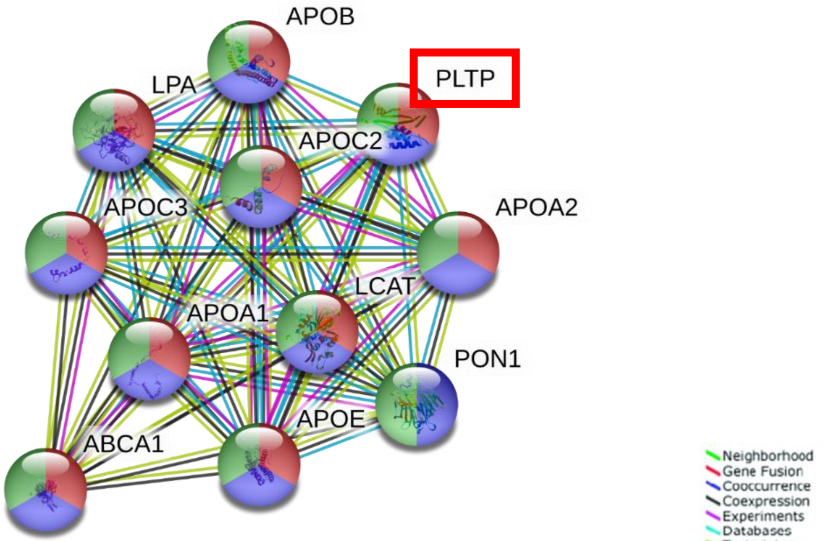

GO-term	description	false discovery rate	
GO:0071827	plasma lipoprotein particle organization	7.52e-23	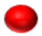
GO:0006629	lipid metabolic process	1.44e-12	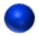
GO:0050789	regulation of biological process	0.0083	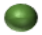

Supplementary Figure S7

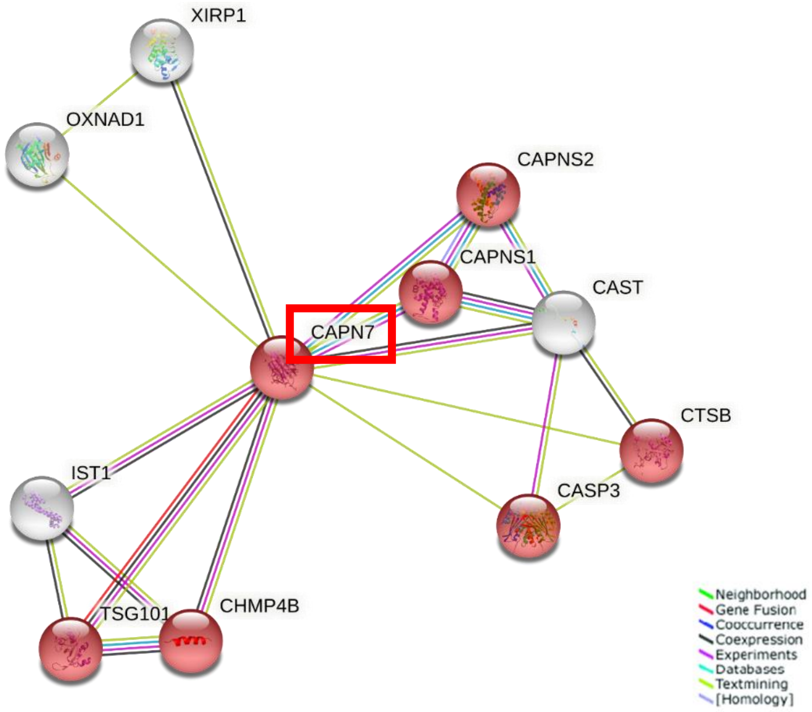

GO-term	description	false discovery rate
GO:0006508	proteolysis	0.00053

Supplementary Figure S8

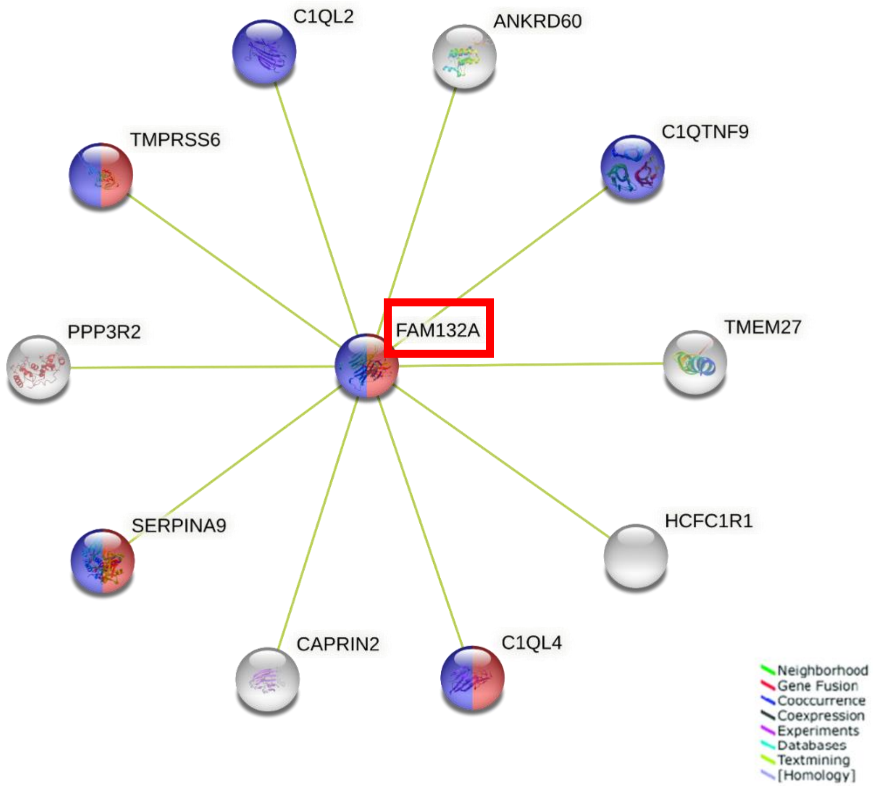

GO-term	description	false discovery rate	
GO:0005576	extracellular region	0.0218	
GO:0005615	extracellular space	0.0341	

Supplementary Figure S9

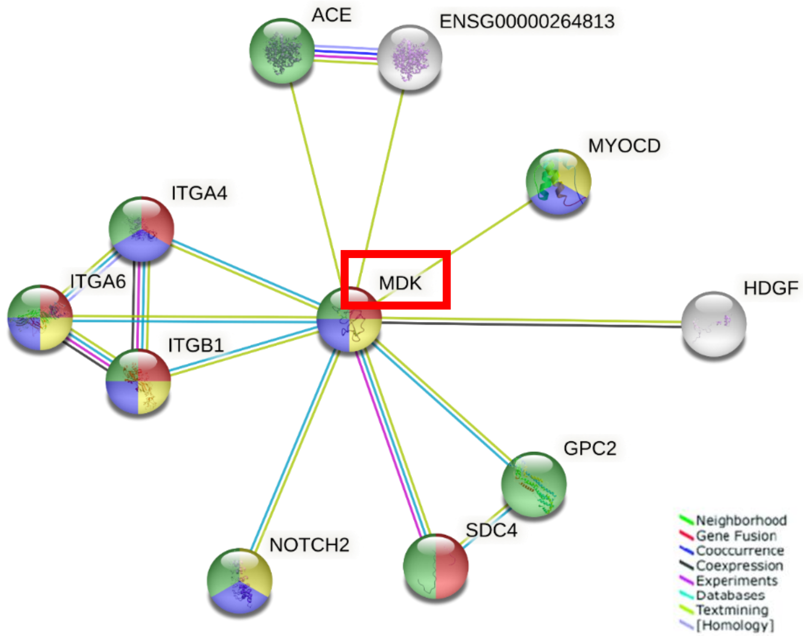

GO-term	description	false discovery rate	
GO:0016477	cell migration	0.0060	red
GO:0043066	negative regulation of apoptotic process	0.0060	blue
GO:0042981	regulation of apoptotic process	0.0060	green
GO:0030154	cell differentiation	0.0060	yellow

Supplementary Figure S10

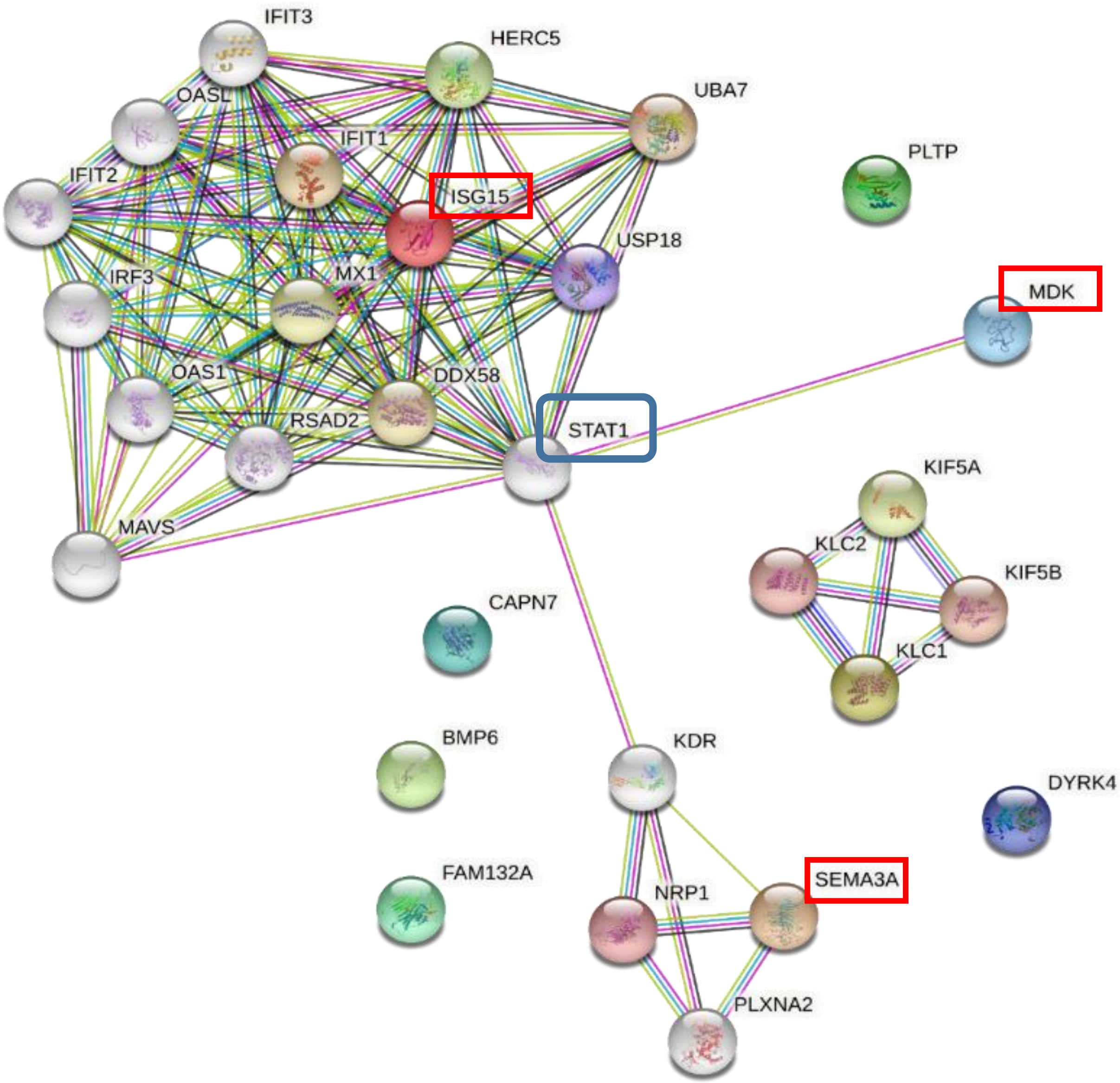

Supplementary Figure S11

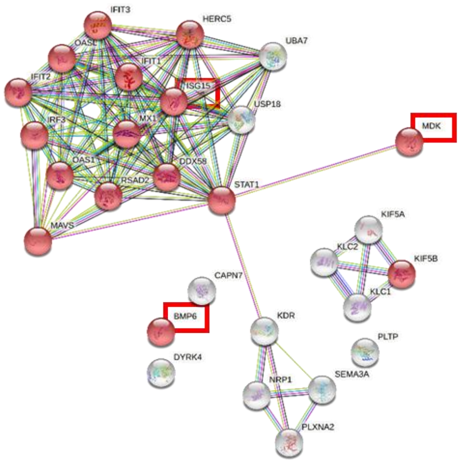

GO-term GO:0006952
Description defense response
false discovery rate 2.04×10^{-10}

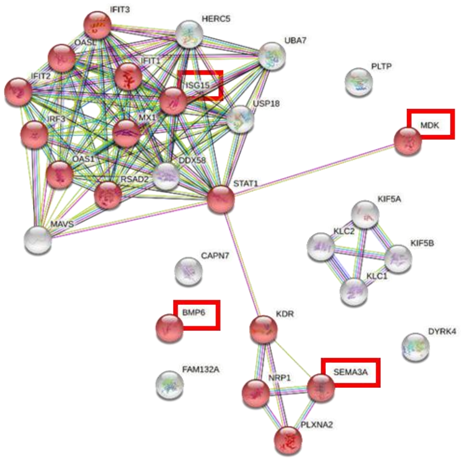

GO-term GO:0007166
Description cell surface receptor signaling pathway
false discovery rate 4.98×10^{-07}

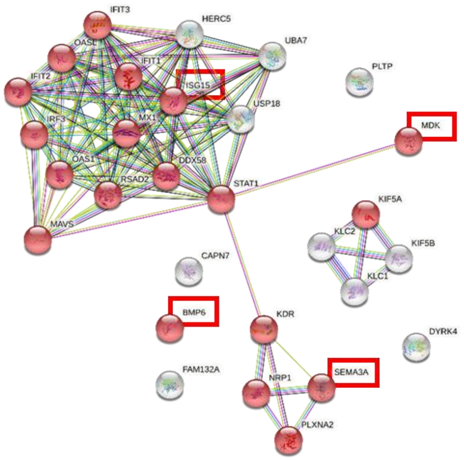

GO-term GO:0023052
Description signaling
false discovery rate 1.98×10^{-04}

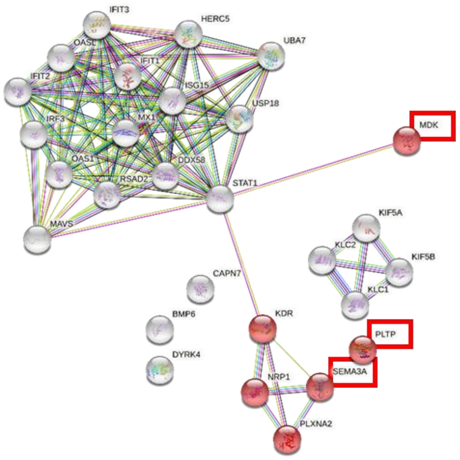

GO-term GO:0048870
Description cell motility
false discovery rate 8.98×10^{-03}

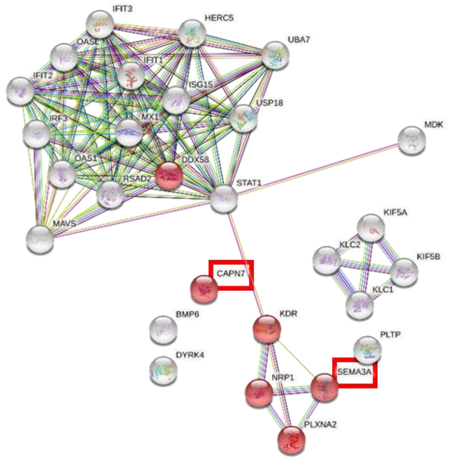

GO-term GO:0030334
Description Regulation of cell migration
false discovery rate 4.2×10^{-03}

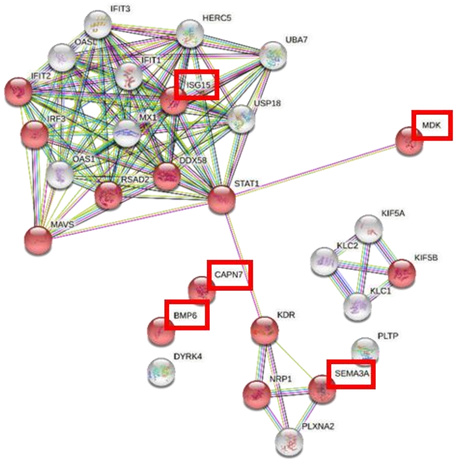

GO-term GO:0048522
Description Regulation of cellular process
false discovery rate 1.73×10^{-02}

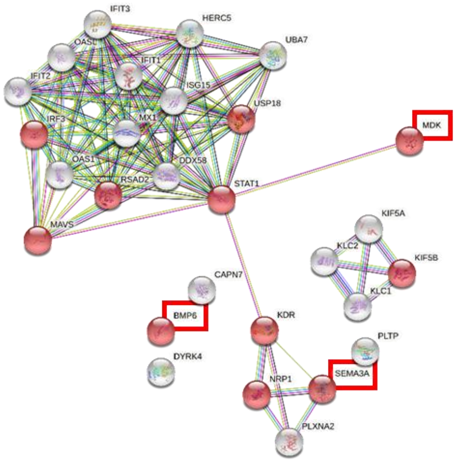

GO-term GO:0010646
Description Regulation of cell communication
false discovery rate 1.89×10^{-02}

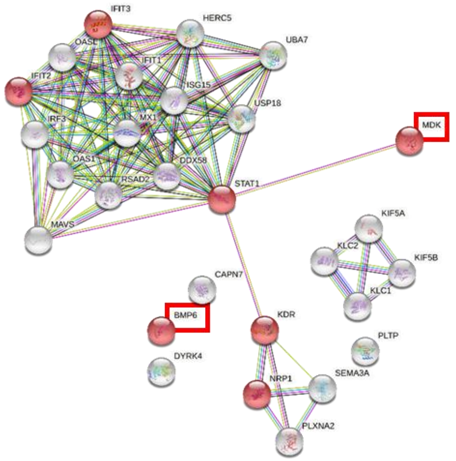

GO-term GO:0042981
Description Regulation of apoptosis process
false discovery rate 1.91×10^{-02}

Supplementary Figure S12

MDK
LLLTLLALL

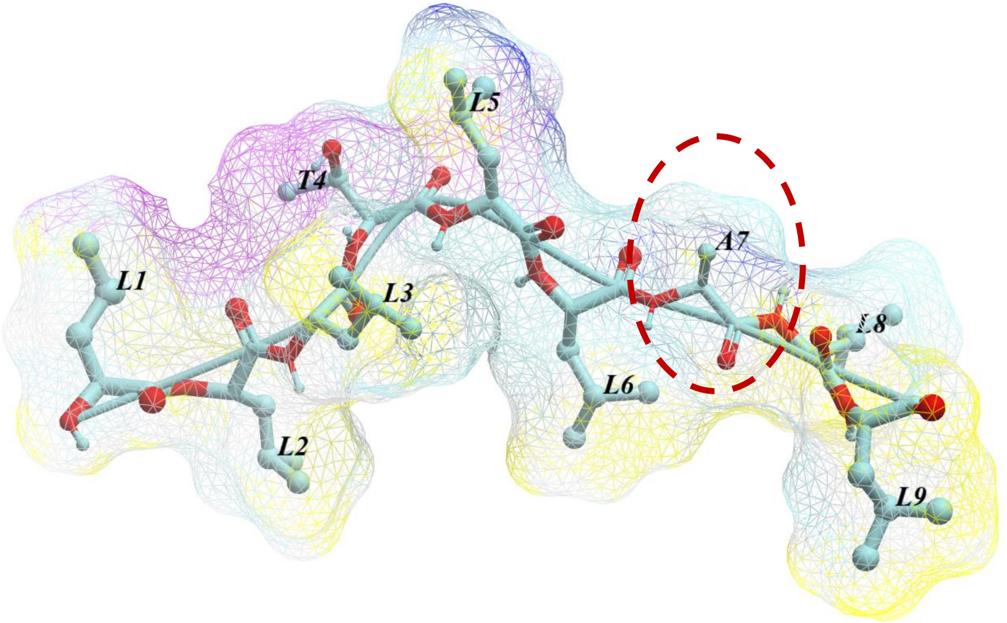

Human Adenovirus
LLLTLLLLL

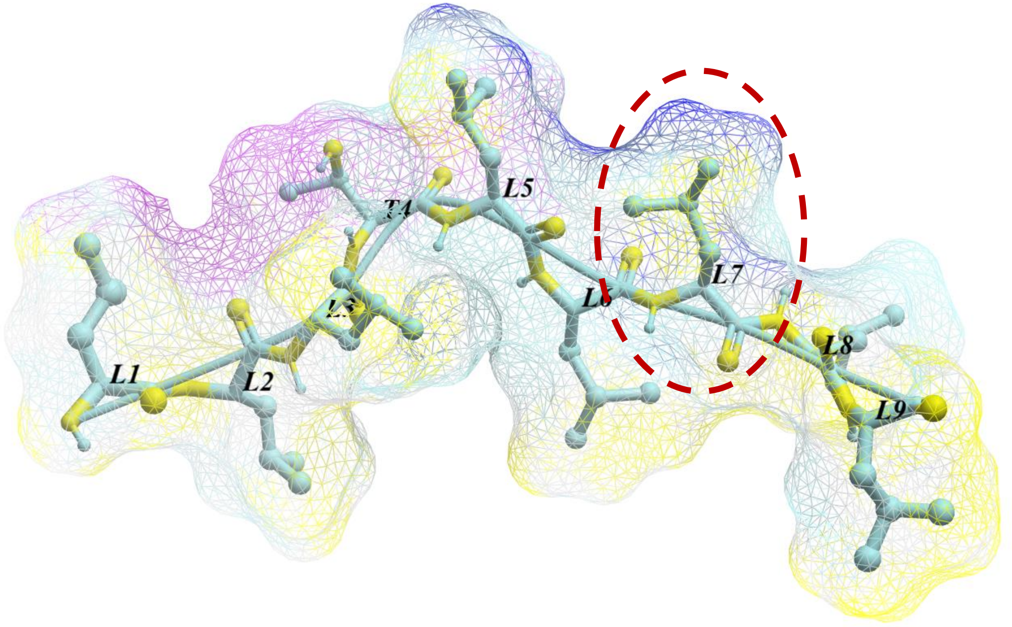

Supplementary Figure S13

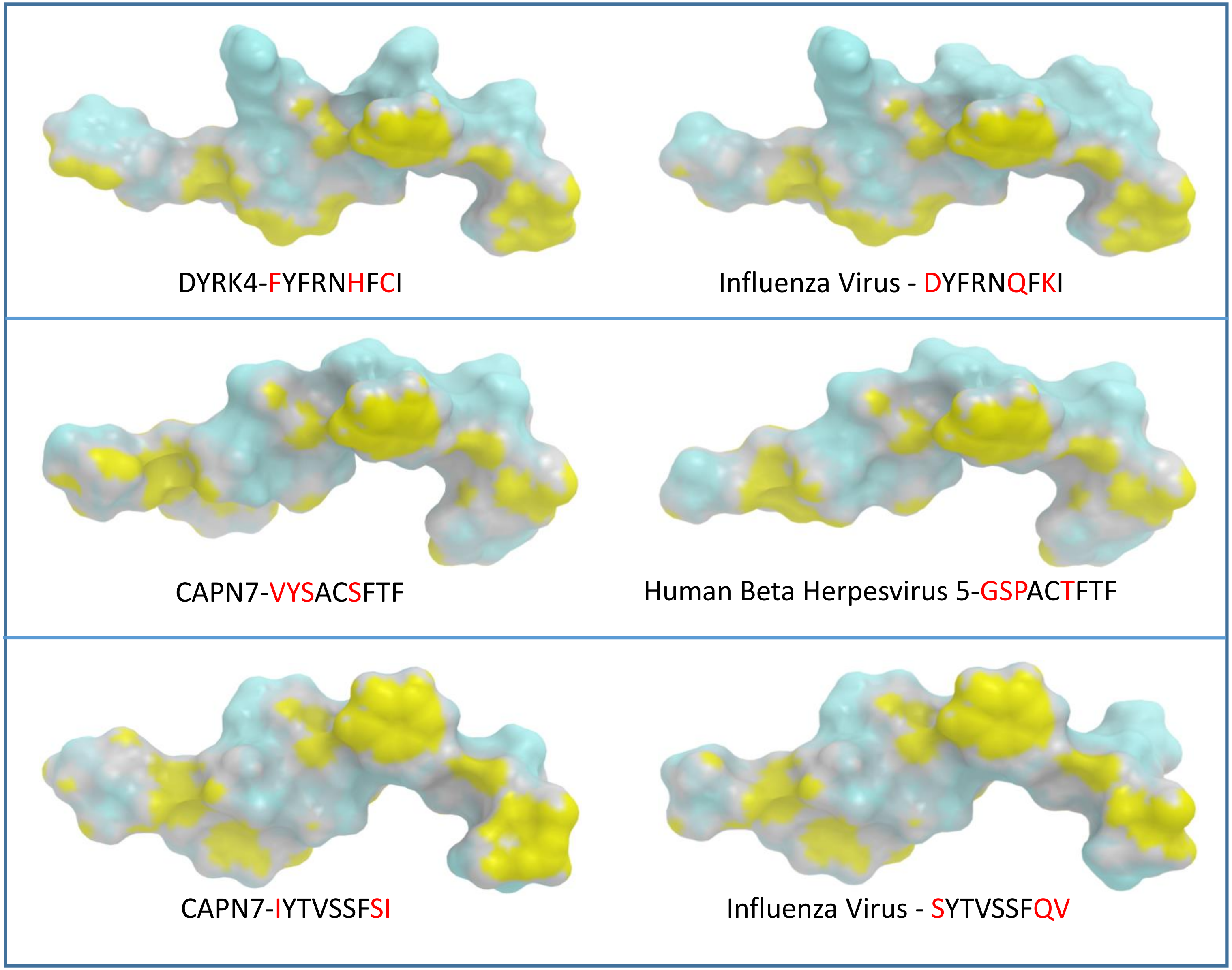

Supplementary Figure S14

DYRK4
FYFRNHFCI

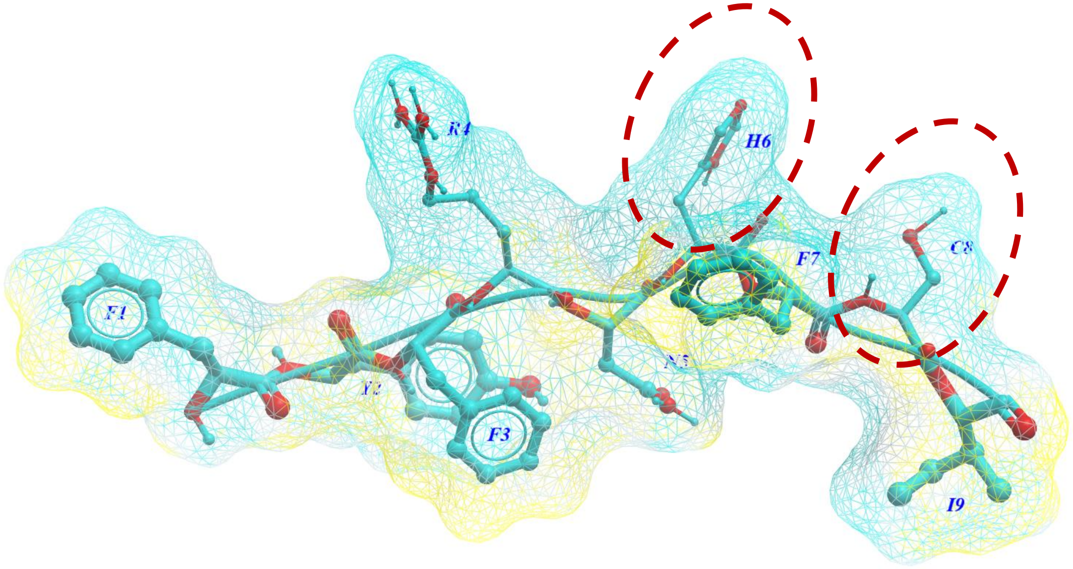

Human Influenza virus
DYFRNQFKI

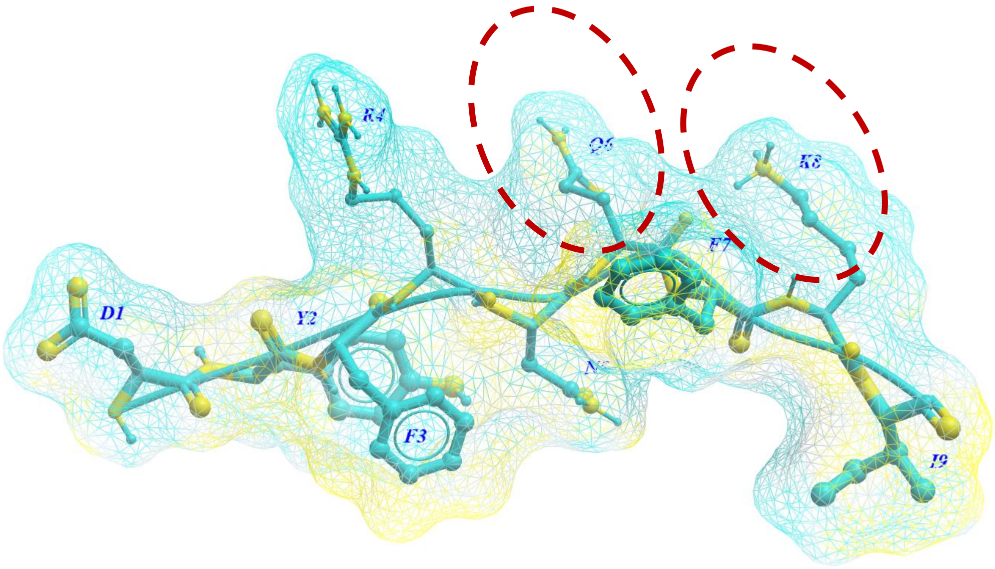

Supplementary Figure S15

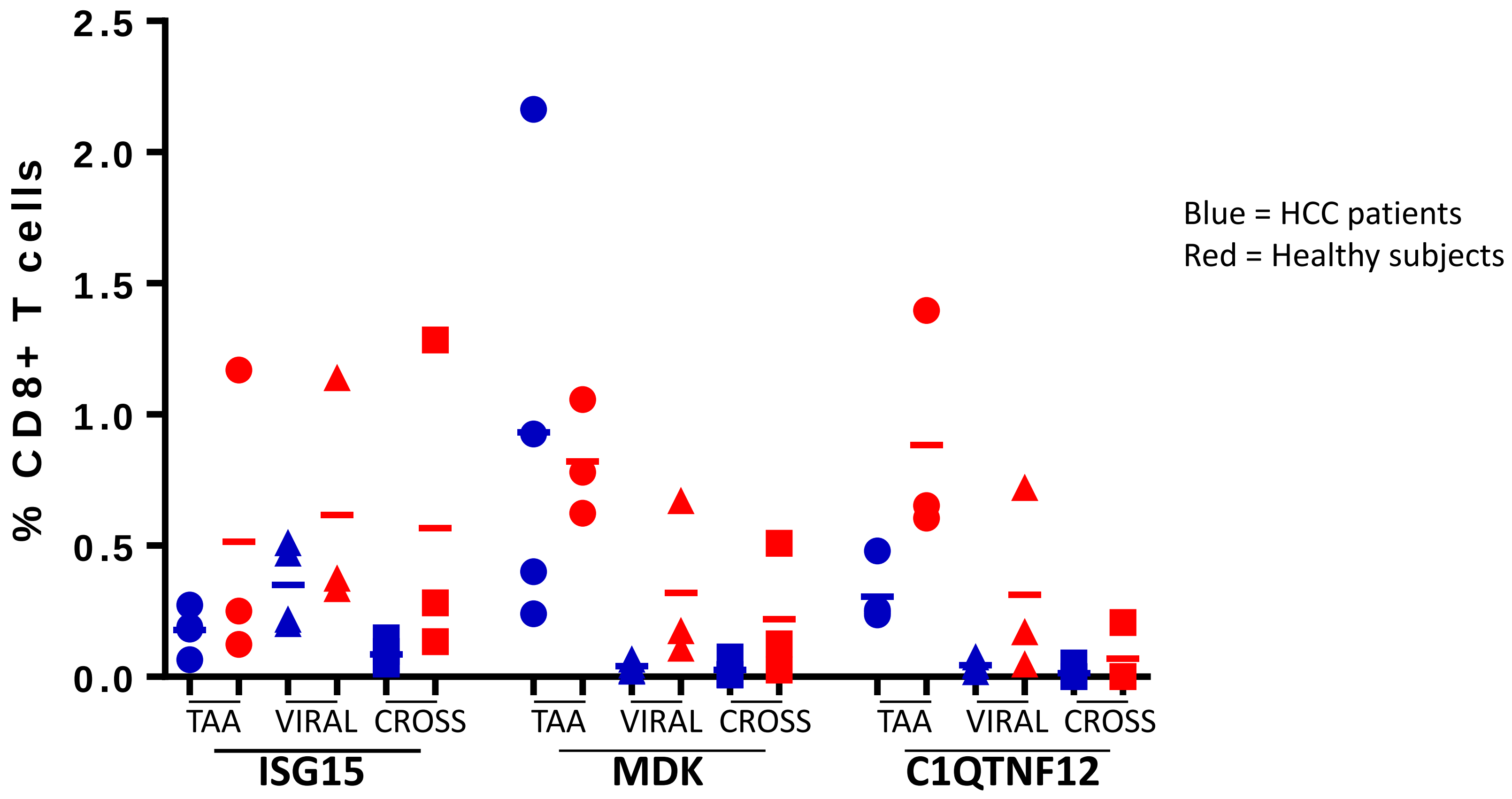

Supplementary Figure S16

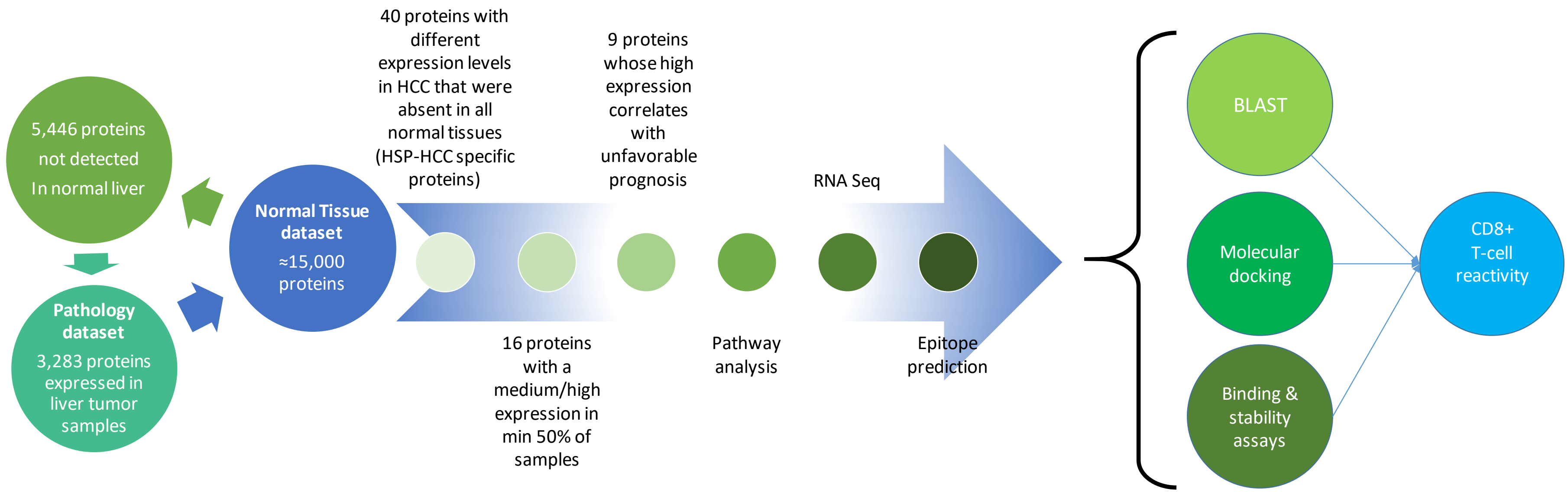

Table S1. HCC-specific proteins (HSP). The listed proteins are expressed at different levels only in HCC samples and no expression is reported in any normal tissue.

Protein name	LIVER CANCER EXPRESSION LEVELS				% of positive samples
	High	Medium	Low	Not detected	
ISG15	9	2	0	0	100%
KLC1	8	3	0	0	100%
CAPN7	7	3	0	2	83%
SERPINF2	6	4	1	1	92%
ORM1	5	5	1	1	92%
ORM2	5	5	1	1	92%
SEMA3A	5	7	0	0	100%
FGF17	5	4	0	3	75%
IL1R2	5	7	0	0	100%
MASP2	5	4	3	0	100%
ANGPT2	4	2	2	3	73%
CLEC3B	4	1	2	2	78%
ADAMTSL2	3	3	2	4	67%
AFP	3	5	3	0	100%
APOC2	3	5	0	4	67%
C4BPA	3	3	0	5	55%
CFB	3	7	1	0	100%
SERPINA7	3	7	1	0	100%
FAM186B	3	3	2	4	67%
BMP6	2	8	0	0	100%
C7	2	9	1	0	100%
CRLF1	2	2	6	2	83%
F2	2	6	2	1	91%
LRG1	2	6	1	1	90%
C1QTNF12	2	2	2	6	50%
MDK	2	1	3	5	55%
APOC1	1	4	2	5	58%
CHRD	1	9	1	1	92%
CP	1	5	6	0	100%
DYRK4	1	11	0	0	100%
ITIH4	1	4	6	1	92%
PLTP	1	10	1	0	100%
PON1	1	5	3	0	100%
TNFRSF1B	1	8	2	0	100%
ADAMTS15	1	7	2	2	83%
KLK14	1	9	2	0	100%
PEBP4	1	4	4	3	75%
PI15	1	2	3	6	50%
RGPD8	1	4	2	4	64%
RSPO2	1	5	4	2	83%

Table S2. Risk factors and clinical stage of HCC patients enrolled for the transcriptomics analyses.

Sample	HCV	HBV	Cirrhosis	EtOH	NASH	Child
**10584**	Yes	Yes	Yes	No	No	Child A
**10594**	Yes	No	No	No	No	n.a.
**10615**	No	No	No	Yes	Yes	(chronic hepatitis)
**10619**	Yes	No	Yes	Yes	No	Child C
**10622**	No	No	Yes	No	No	Child A
**10627**	No	No	Yes	Yes	Yes	Child A
**10628**	Yes	No	Yes	No	No	Child A
**10632**	Yes	No	Yes	No	No	Child A
**10634**	No	No	Yes	Yes	Yes	Child A
**10635**	No	No	Yes	Yes	No	n.a.
**HLA053**	Yes	No	Yes	No	No	n.a.
**HLA058**	No	No	Yes	No	No	n.a.
**HLA063**	Yes	No	Yes	No	No	Child A5
**HLA063**	Yes	No	No	No	No	Child A6
**HLA065**	No	No	Yes	No	No	Child A5
**HLA066**	Yes	No	No	No	No	n.a.
**HLA067**	No	yes	No	No	No	n.a.
**HLA076**	Yes	Yes	No	No	No	n.a.
**HLA078**	Yes	No	Yes	No	No	Child A
**HLA082**	No	Yes	No	No	No	n.a.
**ESTP-3**	No	Yes	No	No	No	n.a.
**ESTP-4**	Yes	No	Yes	Yes	No	Child C
**ESTP-5**	Yes	Yes	No	No	No	n.a.
**ESTP-6**	Yes	No	No	No	No	n.a.

Table S3. STRING analysis for each of the 9 HSP-pp. The most relevant pathways, with a very low false discovery rate value are listed.

PROTEIN NAME	PATHWAY	false discovery rate
**ISG15**	type I interferon signaling pathway	**1.07e-12**
	defense response to virus	**1.27e-12**
	negative regulation of type I interferon production	**2.30e-07**
	regulation of cytokine production	**0.00016**
	regulation of immune system process	**0.00049**
**KLC1**	microtubule-based movement	**1.31e-10**
	antigen processing and presentation of exogenous peptide antigen via MHC class II	**2.30e-07**
	transport	**0.00053**
	vesicle-mediated transport	**0.0019**
	immune system process	**0.0086**
**SEMA3A**	neuron development	**4.0e-14**
	regulation of cellular component movement	**3.14e-08**
	regulation of cell migration	**9.33e-06**
	cell migration	**0.0033**
	epithelium development	**0.0080**
**BMP6**	BMP signaling pathway	**3.45e-14**
	positive regulation of transcription by RNA polymerase II	**4.31e-07**
	cell surface receptor signaling pathway	**2.60e-06**
	regulation of cell differentiation	**6.97e-06**
	regulation of cell population proliferation	**5.71e-06**
**DYRK4**	microtubule severing	**0.0026**
**PLTP**	plasma lipoprotein particle organization	**7.52e-23**
	lipid metabolic process	**1.44e-12**
	regulation of biological process	**0.0083**
**CAPN7**	proteolysis	**0.00053**
**C1QTNF12 (FAM132A)**	extracellular region	**0.0218**
	extracellular space	**0.0341**
**MDK**	cell migration	**0.0060**
	negative regulation of apoptotic process	**0.0061**
	regulation of apoptotic process	**0.0062**
	cell differentiation	**0.0063**

Table S4. Number of strong (SB) and weak (WB) binders predicted for the 9 HSP-pp for HLA-A*02:01 and 24:02 alleles.

PROTEIN	HLA-A	SB	WB
ISG15	HLA-A*02:01	5	8
	HLA-A*24:02	0	3
	**Total**	**5**	**11**
MDK	HLA-A*02:01	3	1
	HLA-A*24:02	0	1
	**Total**	**3**	**2**
KLC1	HLA-A*02:01	9	9
	HLA-A*24:02	4	6
	**Total**	**13**	**15**
CAPN7	HLA-A*02:01	10	20
	HLA-A*24:02	15	27
	**Total**	**25**	**47**
SEMA3A	HLA-A*02:01	5	7
	HLA-A*24:02	8	33
	**Total**	**13**	**40**
BMP6	HLA-A*02:01	5	13
	HLA-A*24:02	3	11
	**Total**	**8**	**24**
C1QTNF12	HLA-A*02:01	5	10
	HLA-A*24:02	2	5
	**Total**	**7**	**15**
DYRK4	HLA-A*02:01	2	11
	HLA-A*24:02	7	18
	**Total**	**9**	**29**
PLTP	HLA-A*02:01	7	22
	HLA-A*24:02	9	16
	**Total**	**16**	**38**
	**CUMULATIVE**	**99**	**221**

Table S5. List of predicted epitopes implemented with prediction of binding stability expressed as half life time in hours (Thalf).

HLA-A*02:01					HLA-A*24:02				
**PROTEIN**	**SEQ**	**AFF**	**Thalf**	**Binder**	**PROTEIN**	**SEQ**	**AFF**	**Thalf**	**Binder**
**ISG15**	***MLAGNEFQV***	**4,35**	**5,22**	**SB**					
	GVQDDLFWL	69,76	0,7	WB					
	ALQDRVPLA	70,13	3,09	WB					
	RLAVHPSGV	82,29	4,73	WB					
	RLTQTVAHL	90,38	6,75	WB					
**MDK**	***ALLALTSAV***	**10,06**	**9,85**	**SB**					
	LLLTLLALL	11,34	4,69	WB					
	FLLLTLLAL	17,89	5,1	WB					
**KLC1**	***TMLNILALV***	**5,14**	**17,22**	**SB**	**KLC1**	***YYYQRALEI***	**12,69**	**4,06**	**SB**
	ALSNHLNAV	**10,73**	**18,84**	**SB**					
	VMMALSNHL	11,55	3,64	WB					
	ILQSLLETL	31,65	4,15	WB					
	KLGPDDPNV	46,49	1,97	WB					
	SLLETLKCL	53,81	3,24	WB					
	KQLNNLALL	62,31	1,95	WB					
	GLSEAQVMM	93,94	1,08	WB					
**CAPN7**	***RQYSVGFEV***	**18,12**	**3,19**	**SB**	**CAPN7**	***VYSACSFTF***	**3,83**	**61,9**	**SB**
	VIIDDQLPV	20,16	4,74	WB		***IYTVSSFSI***	**14,92**	**5,44**	**SB**
	KITEYLERV	**26,12**	**15,36**	**SB**		***IFNIIPSTF***	**32,18**	**3,11**	**SB**
	KIDNGIFWI	28,17	1,75	WB		***YYDVIYLSW***	**53,61**	**5,88**	**SB**
	KQNTIHYTV	84,06	3,03	WB					
**SEMA3A**	AMYNPVFPM	8,47	2,41	WB	**SEMA3A**	VWYRDFMQL	63,51	0,6	WB
	TLLKVTLEV	**10,07**	**5,76**	**SB**		***LYSGTAADF***	**84,34**	**2,2**	**SB**
	KLLTASLLI	13,26	3	WB		***LYACGTGAF***	**91,7**	**2,72**	**SB**
	TMDEFCEQV	23,43	6,96	WB					
	**RIVCLFWGV**	**15,92**	**4,94**	**SB**					
**BMP6**	***FMLDLYNAL***	**3,7**	**3,17**	**SB**	**BMP6**	KQPFMVAFF	43,81	0,57	WB
	MVMSFVNLV	**4,72**	**11,07**	**SB**					
	FLNDADMVM	10,28	2,04	WB					
	FLISIYQVL	**11,1**	**4,82**	**SB**					
**C1QTNF12**	***FQFSASLHV***	**7,76**	**5,7**	**SB**					
	LLHEFQELL	16,18	4,18	WB					
	LLGPQLVLL	37,45	2,61	WB					
	TLLHEFQEL	54,09	1,44	n/a					
**DYRK4**	VILGHPYDV	62,7	1,88	WB	**DYRK4**	***FYFRNHFCI***	**12,53**	**22,36**	**SB**
	SLGCITAEL	31,08	1,72	WB		***VYTYIQSRF***	**22,25**	**13,91**	**SB**
						VHMKDFFYF	**34,95**	**1,96**	**SB**
**PLTP**	***ALFGALFLA***	**4,79**	**7,31**	**SB**	**PLTP**	***VYVAFSEFF***	**7,21**	**14,55**	**SB**
	LLNSLLDTV	**9,24**	**13,38**	**SB**		***TYFGSIVLL***	**60,74**	**2,09**	**SB**
	ALIPLQAPL	21,34	4,21	WB		***LYHAGTVLL***	**68,06**	**2,92**	**SB**
	FLEQELETI	99,88	3,04	n/a		TFITSGMRF	99,93	1,31	WB
	LLNQQICPV	**10,28**	**34,15**	**SB**					
	YINASAEGV	92,99	2,15	WB					

Table S6. Outcome of interrogation of the HLA Ligand Atlas for the identification by mass spectometry of the predicted peptides in normal tissues.

Peptide Sequence	HLA Classes	HLA Alleles	Tissues	Proteins
ALFGALFLA	HLA-1	A*02:01, A*02:05	Adrenal gland, Bladder, Colon, Esophagus, Lung, Ovary, Small intestine, Testis, Thymus	PLTP_HUMAN
ALLALTSAV	no match			
ALSNHLNAV	HLA-1	A*02:01, C*05:01	Ovary	KLC1_HUMAN
FLISIYQVL	no match			
FMLDLYNAL	no match			
FQFSASLHV	no match			
FYFRNHFCI	no match			
IFNIIPSTF	no match			
IYTVSSFSI	no match			
KITEYLERV	HLA-1	A*02:01, C*05:01	Ovary, Thymus, Uterus	CAN7_HUMAN
LLNQQICPV	no match			
LLNSLLDTV	no match			
LYACGTGAF	no match			
LYHAGTVLL	no match			
LYSGTAADF	no match			
MLAGNEFQV	no match			
MVMSFVNLV	no match			
RIVCLFWGV	no match			
RQYSVGFEV	no match			
TLLKVTLEV	no match			
TMLNILALV	no match			
TYFGSIVLL	no match			
VHMKDFFYF	no match			
VYSACSFTF	no match			
VYTYIQSRF	HLA-1	A*23:01, A*24:02, C*04:01, C*06:02, C*07:01, C*07:04	Bladder, Bone marrow, Cerebellum, Esophagus, Heart, Liver, Lung, Muscle, Ovary, Pancreas, Prostate, Small intestine, Spleen, Thymus, Thyroid, Trachea, Uterus	DYRK4_HUMAN
VYVAFSEFF	no match			
YYDVIYLSW	no match			
YYYQRALEI	no match			
